# Leveraging machine learning essentiality predictions and chemogenomic interactions to identify antifungal targets

**DOI:** 10.1038/s41467-021-26850-3

**Published:** 2021-11-11

**Authors:** Ci Fu, Xiang Zhang, Amanda O. Veri, Kali R. Iyer, Emma Lash, Alice Xue, Huijuan Yan, Nicole M. Revie, Cassandra Wong, Zhen-Yuan Lin, Elizabeth J. Polvi, Sean D. Liston, Benjamin VanderSluis, Jing Hou, Yoko Yashiroda, Anne-Claude Gingras, Charles Boone, Teresa R. O’Meara, Matthew J. O’Meara, Suzanne Noble, Nicole Robbins, Chad L. Myers, Leah E. Cowen

**Affiliations:** 1 Department of Molecular Genetics, University of Toronto, Toronto, ON M5G 1M1 Canada; 2 Department of Computer Science and Engineering, University of Minnesota, Minneapolis, MN 55455 USA; 3 Department of Microbiology and Immunology, UCSF School of Medicine, San Francisco, CA 94143 USA; 4 Lunenfeld-Tanenbaum Research Institute, Sinai Health System, Toronto, ON M5G 1X5, Canada; 5 Donnelly Centre, University of Toronto, Toronto, ON M5S 3E1 Canada; 6 RIKEN Center for Sustainable Resource Science, Wako, Saitama 351-0198 Japan; 7 Department of Microbiology and Immunology, University of Michigan Medical School, Ann Arbor, MI 48109 USA; 8 Department of Computational Medicine and Bioinformatics, University of Michigan, Ann Arbor, MI 48109 USA

**Keywords:** Pathogens, Fungal systems biology, Functional genomics, Antifungal agents

## Abstract

Fungal pathogens pose a global threat to human health, with *Candida albicans* among the leading killers. Systematic analysis of essential genes provides a powerful strategy to discover potential antifungal targets. Here, we build a machine learning model to generate genome-wide gene essentiality predictions for *C. albicans* and expand the largest functional genomics resource in this pathogen (the GRACE collection) by 866 genes. Using this model and chemogenomic analyses, we define the function of three uncharacterized essential genes with roles in kinetochore function, mitochondrial integrity, and translation, and identify the glutaminyl-tRNA synthetase Gln4 as the target of N-pyrimidinyl-β-thiophenylacrylamide (NP-BTA), an antifungal compound.

## Abstract (web summary)

The analysis of essential genes in pathogens can be used to discover potential antimicrobial targets. Here, the authors use a machine learning model and chemogenomic analyses to generate genome-wide gene essentiality predictions for the fungal pathogen *Candida albicans*, define the function of three uncharacterized essential genes, and identify the target of a new antifungal compound.

## Introduction

While modern medicine has enabled survival of maladies that would otherwise have been lethal, this has been accompanied by an expansion of immunosuppressed patient populations vulnerable to pathogenic microbes. Among the microbial threats, fungi have emerged as a leading cause of human disease, particularly in immunocompromised individuals^1–3^. These opportunistic invaders include *Candida* species, which are the primary cause of systemic fungal infection in North America, with devastating mortality rates of ~40%^3,4^. While *Candida albicans* is the leading causal agent of these infections, there has been an alarming increase in drug-resistant non-*albicans* species^5–7^. Treatment of these infections is hindered by the limited antifungal armamentarium with only three main drug classes available for clinical use: azoles, polyenes, and echinocandins. Both azoles and polyenes target the membrane sterol ergosterol, with azoles inhibiting its biosynthesis and polyenes extracting the essential sterol from cell membranes^8,9^. Echinocandins inhibit synthesis of β-(1,3) glucan, a key component of fungal cell walls^8,9^. Thus, there is a pressing need to identify additional targets to bolster the antifungal pipeline.

The vast majority of antimicrobial agents in clinical use target functions essential for pathogen viability. This includes azoles and echinocandins, which target the essential gene products Erg11 and Fks1, respectively^8,9^. Essential genes that are fungal-specific or have limited conservation in humans provide attractive targets for selectively killing the pathogen^10^, motivating systematic analysis of genes required for fungal survival to expand the target space for drug development. Functional genomic analyses in the model yeast *Saccharomyces cerevisiae* have established extensive functional redundancy and buffering, as only ~17% of genes in *S. cerevisiae* are essential under standard laboratory conditions^11,12^. Extrapolation to fungal pathogens is limited as ~40% of *C. albicans* genes lack identifiable homologs in *S. cerevisiae* and ~19% of *C. albicans* predicted proteins display no significant similarity to proteins from other organisms^13^.

Functional genomic analysis in *C. albicans* provides unprecedented power for identifying additional therapeutic targets. Traditionally, large-scale genetic studies were hampered by the organism’s diploid genome and lack of a sexual cycle^14–16^. One of the foundational systematic screens focused on identifying genes required for pathogenicity in a mouse and exploited a library of homozygous deletion mutants covering ~11% of the genome^17^. More recently, a stable haploid *C. albicans* isolate was employed to generate a genome-wide transposon mutant collection that was evaluated for gene essentiality^18^. Finally, a functional genomics resource termed the gene replacement and conditional expression (GRACE) collection was generated to define gene function in *C. albicans*^19^. In this mutant library, each strain carries a precise replacement of one of the two alleles of a gene with an auxotrophic marker, while the remaining allele is under the control of a tetracycline-repressible promoter such that gene expression can be repressed by the tetracycline analog doxycycline (DOX)^19^. Prior to this study, the GRACE collection included 2357 strains covering 2327 genes and was enriched for orthologs of essential genes in *S. cerevisiae* or the fission yeast *Schizosaccharomyces pombe*^19^. In vitro screening of the GRACE collection identified 634 strains with a severe or complete growth defect following target gene repression, and established that the predictive value of *S. cerevisiae* essentiality was limited to 52.4%^20^, emphasizing the necessity of defining the essential gene set directly in the pathogen.

Here, we established a machine-learning model to provide a comprehensive essentiality prediction for the ~6500 genes annotated in the *C. albicans* genome^21^. Predictions from this model guided the generation of 866 additional GRACE mutants (termed GRACEv2), including 115 strains for predicted essential genes, further expanding this functional genomics resource to provide ~48% coverage of the *C. albicans* genome. By coupling our computational prediction with this expanded GRACE collection, we identified 149 fungal-specific essential genes. Integrating our machine-learning model with phylogenetic analysis and co-expression datasets, we described the function of several previously uncharacterized genes involved in kinetochore function (*C1_01070C* or *KRP1*), mitochondrial integrity (*C6_03200W* or *EMF1*), and translation initiation (*C2_04370W* or *TIF33*). Finally, we identified N-pyrimidinyl-beta-thiophenylacrylamide (NP-BTA) as a molecule with potent activity against *C. albicans* that inhibits the essential glutaminyl-tRNA synthetase Gln4. Collectively, this work leverages machine learning, functional genomics, and chemical genomics to characterize essential genes and defines additional targets to advance antifungal drug development.

## Results

### Leveraging a machine-learning model to generate genome-wide predictions of *C. albicans* gene essentiality

**Fig. 1:**
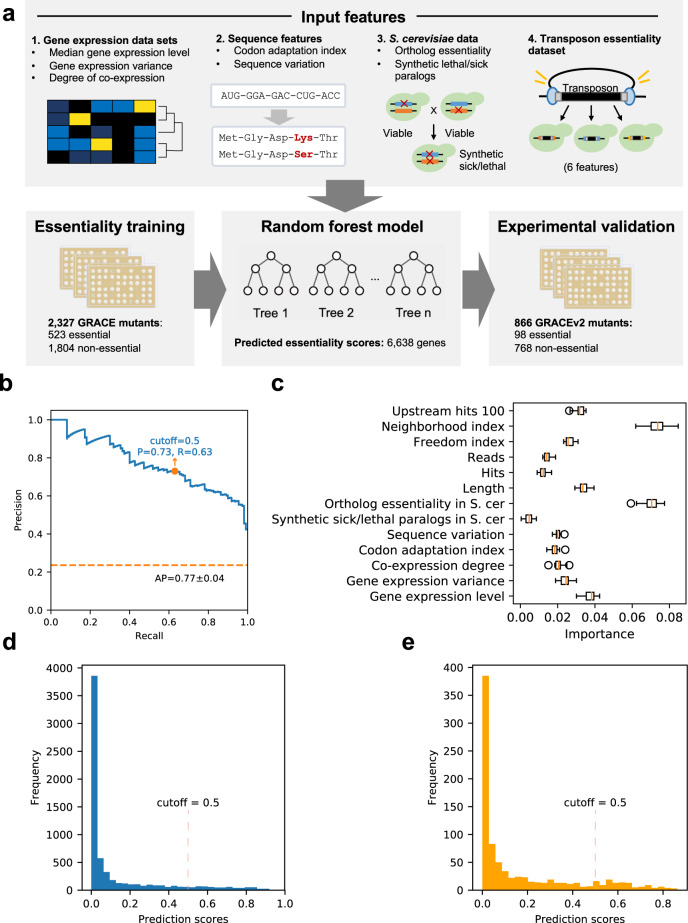
Building a machine-learning model to predict essentiality and testing on the original GRACE collection. **a** Overview of the input, output, and validation process of our random forest model. **b** Precision-recall curve of our random forest model on 20% of the GRACE gene set. The model was trained and optimized on the other 80% of the GRACE gene set. The default stringent cutoff score for essential gene predictions results in a precision of 0.73 and a recall of 0.63, with an average precision score of 0.77. The error bars reflect the standard deviation across estimates derived from 10,000 different resamplings (with replacement) of the test set. **c** Permutation feature importance of our random forest model for the whole GRACE gene set. The decrease in a model upon permutation of that feature score reflects importance, and the box plots show variation for each feature’s importance across 30 permutations. The whiskers extend out to 1.5 times the inter-quartile range, and the flier points reflect outliers beyond 1.5 times the inter-quartile range. S. cer represents *S. cerevisiae*. **d** Distribution of our random forest prediction scores across 6638 *C. albicans* genes. **e** Distribution of prediction scores for the 866 selected candidates for further experimental validation. Source data are provided as a Source Data file.

In order to generate a comprehensive assessment of genes with evidence for essentiality in *C. albicans*, we first collected a set of functional genomic features for use in training a machine-learning model (Supplementary Data 1). These included features derived from a collection of gene expression datasets such as gene expression level (Transcripts Per Kilobase Million (TPM) median), gene expression variance, and degree of co-expression (number of partners in a co-expression network)^22^. It also encompassed a codon adaptation index (CAI), which measures the bias in codon usage across each gene^21^; the number of SNPs per nucleotide for each gene across a set of sequenced *C. albicans* strains^23^; the presence of an essential ortholog in *S. cerevisiae*^12^; and the presence of a duplicated set of paralogs in *S. cerevisiae* that exhibited a synthetic sick/lethal genetic interaction^24^. Finally, we incorporated six features from a recent transposon mutagenesis (TnSeq) study in a stable haploid background for which a previous machine-learning model was developed^18^ (Fig. 1a).

With this collection of features, we built a supervised machine-learning model based on random forests (RF)^25^ using the *C. albicans* GRACE mutant library as a gold standard for gene essentiality^19^. We confirmed the essentiality of all GRACE strains by pre-treating mutants with a high concentration (100 µg/mL) of DOX before transfer onto solid medium containing 100 µg/mL DOX and allowing cells to grow for 48 h. Colony images were qualitatively scored for growth defects using a scale from 0 (growth comparable to wild type) to 4 (no growth) by two independent researchers (Supplementary Fig. 1a and Supplementary Data 2). A gene was classified as essential if its corresponding GRACE strain was able to grow robustly in the absence of DOX but displayed a severe growth defect in the presence of DOX. In instances where discrepant results were obtained between researchers, essentiality was further characterized by spot dilution assays (Supplementary Fig. 1b). This rigorous assessment ensured that an essential designation was restricted to genes for which depletion resulted in very little to no growth (score of 3 or 4), ensuring that genes for which transcriptional repression merely resulted in modest growth defects (score of 1 or 2) were not falsely identified. Scores were also assigned to strains with growth defects that did not meet essentiality criteria (Supplementary Data 2). While this strict approach may have resulted in the omission of genes previously characterized as essential^26,27^, this assessment of essentiality provided a robust training set for our machine-learning model, which included 523 confirmed essential genes and 1804 non-essential genes. We trained our RF classifier on 80% of this GRACE training set with fivefold cross-validation for hyperparameter tuning, and then tested the performance on the remaining 20%. We achieved an average precision (AP) of 0.77 and an area under the receiver operating characteristic curve (AUC) of 0.92 (Fig. 1b and Supplementary Fig. 2b). Using a standard permutation-based approach^25^, we evaluated the relative importance of features contributing to the model’s performance (Fig. 1c). This highlighted ortholog essentiality in *S. cerevisiae* and neighborhood index measure from the TnSeq screen^18^ as two of the most important features, although all included features contributed unique information (Fig. 1c, Supplementary Note 1 and Supplementary Table 1).

One defining strength of our machine-learning model is that it predicts essentiality for the vast majority of genes annotated in the *C. albicans* genome (6638 gene annotations were included in the model output; see “Methods” for details), making this the most comprehensive essentiality prediction dataset to date. Specifically, it includes predictions for 745 genes that were not addressed in other studies^18^. Using a stringent cutoff of essentiality (RF output score > 0.5), our model predicted 654 genes in *C. albicans* to be essential (Fig. 1d and Supplementary Data 1). These were enriched in diverse processes including ribosome biogenesis, cellular metabolic processes, translation, RNA processing, transcription, mitotic cell cycle, and microtubule cytoskeleton organization (FDR < 1%; Supplementary Data 3).

### Experimental validation of *C. albicans* essentiality predictions

**Fig. 2:**
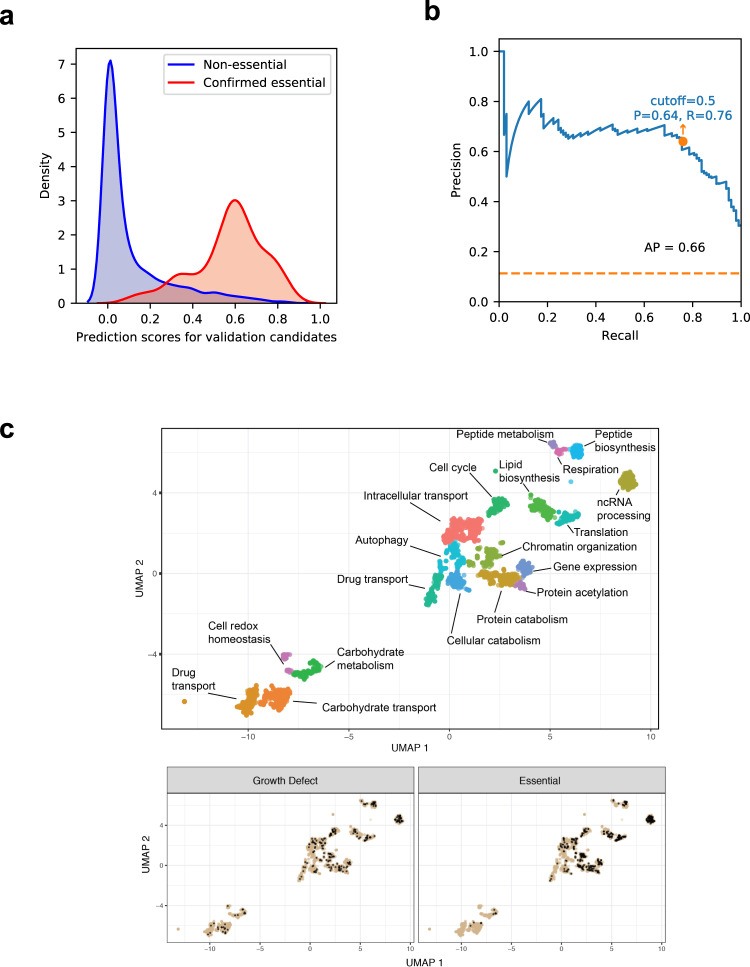
Testing the accuracy of the prediction model with the GRACEv2 collection. **a** Distribution of prediction scores for the 98 experimentally confirmed essential genes and 768 non-essential genes from the validation candidates (GRACEv2 strains). **b** Precision-recall curve of the random forest model derived from the whole GRACE set and tested on the GRACEv2 experimental validation set. The default stringent cutoff score for essential gene predictions results in a precision of 0.64 and a recall of 0.76, with an average precision score of 0.66. **c** Essential genes are enriched in specific functional clusters. Clusters were generated by UMAP embedding of co-expression and functional enrichment was determined by GO term analysis. Source data are provided as a Source Data file.

To assess the accuracy of our prediction model, we selected 866 genes for validation, covering the range of RF prediction scores (Figs. 1e and 2a), including 115 genes with RF scores >0.5. For each selected gene, we constructed a corresponding GRACE strain, expanding the genome coverage of this functional genomics resource from 2327 to 3193 mutants or 48% of the *C. albicans* genome (GRACEv2 collection). We experimentally tested the essentiality of the GRACEv2 collection and for genes with an RF prediction score of >0.5, we confirmed essentiality of the corresponding mutants at a rate of ~64% (74 out of 115) relative to a genome-wide expectation of ~20%^12,18,20^. Of the remaining 41 genes predicted but not confirmed to be essential, 23 showed a slow growth phenotype (growth score of 1 or 2). Among the 866 strains tested in the GRACEv2 collection, 98 genes were confirmed as essential (Supplementary Data 1). We found that the RF prediction score was highly effective in identifying essential genes, resulting in an AP of 0.66 and AUC of 0.95 on the GRACEv2 validation set (Fig. 2b and Supplementary Fig. 2c).

These assessments confirmed that use of a machine-learning model that robustly integrates numerous data sources substantially improves the accuracy of essential gene prediction over models based on individual features. For example, essentiality of orthologs in *S. cerevisiae* predicts essentiality in *C. albicans* with only 36% accuracy (or 46.7% accuracy if one includes *C. albicans* mutants with slow growth phenotypes), and synthetic lethality of duplicated paralogs in *S. cerevisiae* predicts essentiality with 31% accuracy (Supplementary Data 1). Also, these analyses allowed us to assess essentiality phenotypes of 43 genes (21 strains from the GRACE collection and 22 strains from the GRACEv2 collection) that were not predicted or assessed for essentiality in previously published models^18^, highlighting the comprehensive nature of our analyses.

Finally, our comprehensive analysis enabled the discovery of additional essential genes. Specifically, when defining known essential genes as the 1278 *C. albicans* genes currently marked as inviable in the *Candida* Genome Database^21^, our set of 621 experimentally confirmed essential genes from the GRACE and GRACEv2 collections included 53 essential genes that were not previously classified as essential. In terms of our essential gene predictions, at a stringent confidence cutoff (RF score > 0.5, estimated precision > 90%), our model covered 46% of the known essential genes and yielded 66 predicted essential genes that were not previously reported as essential. At a more relaxed, but still high confidence cutoff (RF score > 0.15, estimated precision > 70%), we covered 88% of the known essential genes and identified 478 predicted essential genes that were not previously reported (Supplementary Fig. 2d).

We utilized co-expression clustering analysis to determine whether any biological processes are enriched among essential genes in the *C. albicans* genome. Using a non-linear dimensionality reduction, we clustered *C. albicans* genes based on co-expression^22^ (Fig. 2c) and used GO term enrichment of the genes in each cluster to assign a putative function. We then tested each cluster for enrichment of essential genes using the hypergeometric test and found enrichment at the 0.05 significance level for four clusters: translation (120 of 198 genes; *p* < 8e−40), ncRNA processing (123 of 299 genes; *p* < 8e−27), chromatin organization (58 of 229 genes; *p* < 0.02), and peptide biosynthesis (34 of 122 genes; *p* < 0.02).

Genes that are essential in fungi but not present in humans represent priority targets for antifungal drug development, as there is a reduced likelihood of off-target toxicity^26,28^. Of the 621 validated *C. albicans* essential genes, 149 lacked human homologs (Supplementary Data 4). These genes are enriched in diverse processes including organic substance biosynthetic processes, cellular component organization, gene expression, and translation (FDR < 1%; Supplementary Data 3). We paired our findings with drug target databases including DrugBank^29^, Therapeutic Target Database^30^, and PubChem^31^ to identify 97 compounds targeting nine of our experimentally confirmed essential or predicted essential genes, three of them lacking human orthologs (Supplementary Data 5).

### Characterization of Krp1 as a member of the kinetochore

We next leveraged our essentiality predictions to characterize the role of fungal-specific essential genes that lack annotated functions. We identified those genes without predicted *S. cerevisiae* orthologs using the *Candida* Genome Database^21^. Of the 149 fungal-specific essential genes, only four lacked *S. cerevisiae* orthologs: *C1_01070C, C1_09670C, C6_03200W*, and *C2_07260C* (Supplementary Data 4). We initially focused our efforts on *C1_01070C*, which we named *KRP1* (kinetochore-related protein 1) and used BLASTp and conserved domain searches to explore potential gene function. Although this gene was previously annotated in CGD as essential, it remained completely uncharacterized, motivating us to further describe its biological function. These analyses predicted that *KRP1* may localize to the MIND (Mis12/Mtw1-Nnf1-Nsl1-Dsn1) complex to regulate chromosome segregation and cell cycle progression. Notably, no *C. albicans* Dsn1 homolog has been identified to date^21^. In *S. cerevisiae*, the MIND kinetochore subcomplex binds to DNA and recruits remaining assembly complexes, including the DASH/Dam1 complex, which connects microtubules with centromeres during cell division^32^. Although members of the DASH/Dam1 complex localize to kinetochores and regulate proper mitotic chromosome segregation in *C. albicans*^33^, a complete characterization of all ten predicted complex members has yet to be achieved.

**Fig. 3:**
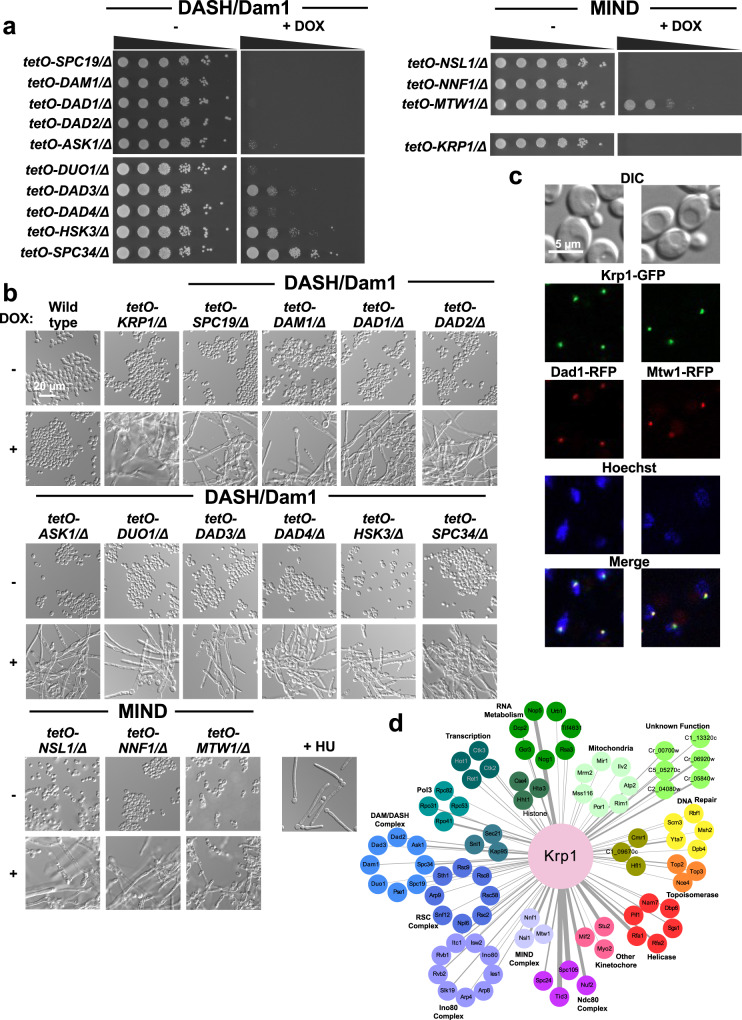
Characterization of Krp1 as a member of the kinetochore complex. **a** Testing the essentiality of kinetochore components. Strains were grown overnight in the absence or presence of 0.05 μg/mL doxycycline (DOX) at which point they were spotted in tenfold dilutions (starting from an OD_600_ of 0.5) onto YNB agar alone or supplemented with 50 μg/mL DOX. Plates were photographed after growth for 48 h at 30 °C. **b** Examining the impact of kinetochore-related genes on *C. albicans* morphology. Strains were grown overnight as described in (**a**). Strains were subsequently subcultured to an OD_600_ of 0.1 in YPD in the absence or presence of 0.05 μg/mL DOX as indicated. The wild-type strain in the absence of DOX was treated with 25 mM hydroxyurea (HU) as indicated. Cultures were incubated at 30 °C for 24 h for GRACE strains or 6 h for HU treatment before cells were visualized by microscopy. Experiment was performed in biological duplicate with similar results. **c** Krp1 localizes to the kinetochore. Strains were subcultured to an OD_600_ of 0.1 in YPD and allowed to grow for 4 h before visualization. Krp1 (green), Dad1 (red), Mtw1 (red), and nuclei (blue) were visualized by fluorescence microscopy. Experiment was performed in biological duplicate with similar results. **d** AP-MS of affinity-tagged Krp1 identified physically interacting proteins. Cells were grown in YPD at 30 °C, and statistically significant interactions were defined through SAINTexpress analysis compared with an unrelated tagged protein. Nodes are grouped and colored based on GO term annotation. The weight of the edges reflects the fold-change in peptide count of Krp1 relative to an unrelated tagged protein (Eno1) for those interacting partners with a BFDR < 1%. Source data are provided as a Source Data file.

To characterize the function of the *C. albicans* DASH/Dam1 complex, MIND complex, and Krp1, we examined essentiality of all known components using spot dilution assays. This was of particular interest, because although in *S. cerevisiae* all members of the DASH/Dam1 and MIND complexes are essential, they are all dispensable for growth in *S. pombe*^34–37^. Consistent with previous studies in *C. albicans*^33,38^, we found that the DASH/Dam1 complex members *SPC19, DAM1, DAD1, DAD2*, and *ASK1* were essential for growth, as well as the previously uncharacterized subunit genes *DUO1* and *DAD4* (Fig. 3a and Supplementary Fig. 3a). Interestingly, *DAD3, HSK3*, and *SPC34* were non-essential, as substantial growth was observed despite transcriptional repression of the target gene with DOX (Fig. 3a and Supplementary Fig. 3). MIND complex members *NNF1* and *NSL1* were essential, along with *KRP1*, whereas depletion of *MTW1* still allowed for growth on agar medium with a high concentration of DOX (Fig. 3a and Supplementary Fig. 3b). This is in contrast to previous work that showed *MTW1* to be essential in *C. albicans*^39^.

We explored the impact of the DASH/Dam1 and MIND components on *C. albicans* morphology, as perturbation of the cell cycle often induces filamentation^40,41^, and proper function of the kinetochore is required for G_2_/M progression^33^. Transcriptional repression of all subunits of the DASH/Dam1 complex with a low concentration (0.05 μg/mL) of DOX induced filamentation in the absence of an inducing cue without significantly impacting growth, similar to the cell cycle inhibitor hydroxyurea (Fig. 3b). Similarly, the depletion of MIND complex members *MTW1, NSL1*, and *NNF1* along with *KRP1* induced filamentation (Fig. 3b).

To test whether Krp1 may function at the kinetochore, we monitored its localization using fluorescence microscopy. Both alleles of *KRP1* were C-terminally tagged with a GFP epitope in a strain with both alleles of either a DASH/Dam1 complex member or MIND complex member C-terminally tagged with RFP (Dad1-RFP or Mtw1-RFP, respectively). Tagging both alleles confirmed functionality, as strains were viable and grew as yeast under non-filament inducing conditions (Fig. 3c). We observed green puncta at the nuclear periphery, which co-localized with red puncta in both strain backgrounds (Fig. 3c), suggesting that Krp1 may directly or indirectly interact with Dad1 and/or Mtw1. Finally, to elucidate the Krp1 interactome in *C. albicans* we performed affinity purification of Krp1-GFP using a GFP-trap resin, followed by mass spectrometry. To distinguish significant interactors from background, we compared interactors identified with Krp1-GFP with those identified with an Eno1-GFP control using SAINTexpress and a Bayesian false discovery rate (BFDR) cutoff of 1% (Fig. 3d and Supplementary Data 6). While Krp1 interacted with many proteins, there was a significant enrichment of interactions with proteins involved in chromosome organization, mitotic cell cycle process, and kinetochore assembly (Supplementary Data 3). Interactions were confirmed with eight out of ten DASH/Dam1 complex members and all members of the MIND complex (Fig. 3d and Supplementary Data 6). Thus, *KRP1* encodes an essential protein that regulates cell cycle progression, likely through its involvement at the kinetochore.

### Leveraging co-expression data to define the function of mitochondrial-related gene *EMF1*

**Fig. 4:**
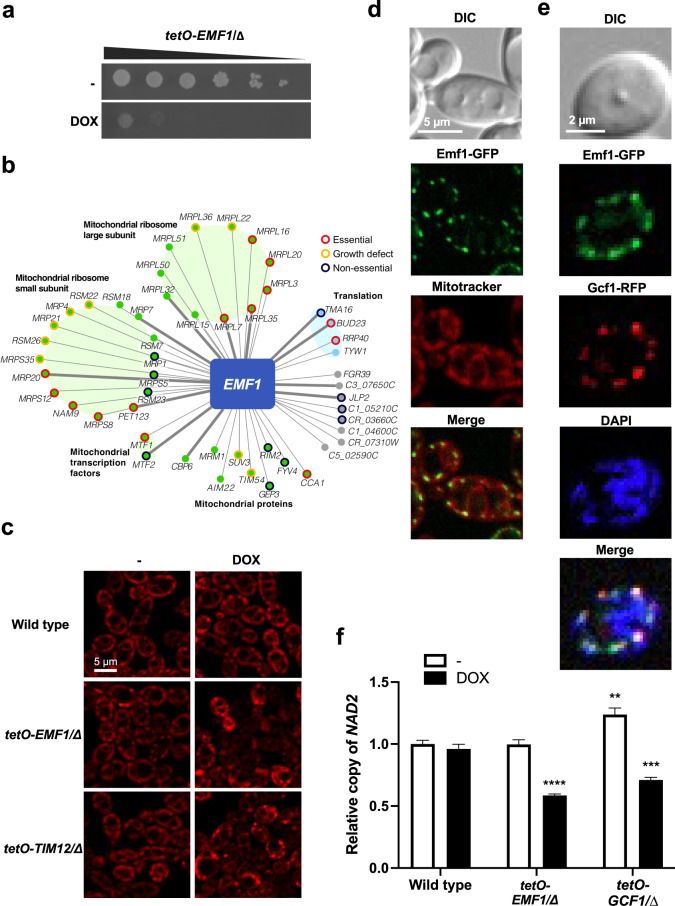
Characterization of Emf1 as a mitochondria component. **a** *C6_03200W* (renamed *EMF1*) is an essential *C. albicans* gene. The *tetO-EMF1/emf1∆* strain was grown and assessed for essentiality as described in Fig. 3a. **b** A co-expression network for *EMF1* identifies multiple mitochondrial proteins in the top 50 co-expressed genes. Nodes represent genes, and edges represent the strength of the co-expression. All genes had a co-expression score of at least 0.997. Green indicates mitochondrial annotation, light blue indicates translation annotation, gray indicates no GO term annotation available. **c** Depletion of *EMF1* perturbs mitochondrial morphology. Strains were grown overnight in the absence or presence of 0.05 µg/mL DOX, subcultured to an OD_600_ of 0.1 with the same respective DOX conditions, and grown for 3 h at 30 °C. Cultures were further incubated with 50 nM Mitotracker Red for 40 m, washed, and resuspended in PBS. MitoTracker Red staining was imaged with the DsRed channel with equal exposure among samples. Experiment was performed in biological duplicate with similar results. **d** Emf1 localizes to the mitochondria. Cells were subcultured to an OD_600_ of 0.1 and allowed to grow for 4 h before visualization. As indicated, cultures were incubated with 50 nM Mitotracker Red for 40 m, washed, and resuspended in PBS. Emf1 (green) and mitochondria (MitoTracker, red) were visualized by fluorescence microscopy. Experiment was performed in biological duplicate with similar results. **e** Emf1 co-localizes with Gcf1 at DAPI-stained mitochondrial nucleoids. Cells were subcultured to an OD_600_ of 0.1 and grown for 4 h before being washed and resuspended in PBS, then incubated with 1 µg/mL DAPI for 1 h. Emf1 (green), Gcf1 (red), and DNA (DAPI, blue) were visualized by fluorescence microscopy. Experiment was performed in biological duplicate with similar results. **f** Transcriptional repression of *EMF1* causes a significant reduction in mtDNA copy number. Relative *NAD2* copy number in wild-type, *tetO-EMF1/emf1∆*, and *tetO-GCF1/gcf1∆* cells in the absence and presence of 0.05 µg/mL DOX as determined by qPCR, using *ACT1* and *GPD1* for normalization. Values shown are relative *NAD2* copy number compared to the wild-type strain in the absence of DOX. Error bars represent SEM above and below the mean of technical triplicates (two-way ANOVA, Bonferroni correction for multiple comparisons, **P < 0.002; ***P < 0.0004; ****P < 0.0001 compared to wild-type untreated). Experiment was performed in biological duplicate with similar results. Source data are provided as a Source Data file.

To elucidate the function of another previously unannotated fungal-specific essential gene, we turned to *C6_03200W*. As observed by spot dilution assays, transcriptional repression of *C6_03200W* with DOX abrogated growth, confirming it as an essential gene (Fig. 4a and Supplementary Fig. 3c). The function of *C6_03200W* could not be predicted using BLASTp or conserved domain searches. Thus, we leveraged the *C. albicans* co-expression dataset used in our machine-learning model, as co-expression partners are predicted to have related functions^22^. *C6_03200W* co-clustered with mitochondrial genes, especially those involved in mitochondrial translation (Fig. 4b), and therefore we named this gene *EMF1* (essential mitochondrial function 1). The mitochondrial localization prediction software Predotar v1.04 supported these findings^42,43^. To determine if *EMF1* governs mitochondrial integrity, we used the mitochondrial dye MitoTracker Red to assess alterations in mitochondrial morphology upon transcriptional repression of this target gene. Depletion of *EMF1* caused the mitochondria to coalesce and lose their tubular morphology, similar to what was observed with depletion of the well-characterized mitochondrial import gene *TIM12* (Fig. 4c).

Next, we epitope-tagged both copies of *EMF1* with GFP and monitored its localization. Tagging both alleles of *EMF1* confirmed functionality, as strains were viable. Emf1-GFP co-localized with MitoTracker Red, forming discrete puncta in a similar pattern to what has been previously reported with the *C. albicans* mitochondrial DNA (mtDNA)-binding protein Gcf1 (Fig. 4d)^44^. To determine if Emf1 co-localizes with Gcf1, we C-terminally tagged both alleles of *GCF1* with RFP in the strain harboring Emf1*-*GFP, and observed Emf1 and Gcf1 co-localized at DAPI-stained mitochondrial nucleoids, densely packed structures of mtDNA and associated proteins (Fig. 4e). Subsequent sequence analysis using the protein structure prediction program Phyre2 suggested Emf1 contains an HMG-box similar to the mitochondrial transcription factor A (TFAM), consistent with a putative role in binding mtDNA. As mtDNA-binding proteins are required to maintain mtDNA copy number^45,46^, we performed quantitative PCR to monitor changes in DNA abundance upon transcriptional repression of *EMF1*. Similar to *GCF1*, transcriptional repression of *EMF1* with a low concentration of DOX caused a significant reduction in the mtDNA-encoded gene *NAD2* relative to the nuclear genes *ACT1* and *GPD1* (Fig. 4f). Together, this suggests Emf1 may act as a mtDNA-binding protein in *C. albicans*.

### Tif33 is a member of the translation initiation complex

**Fig. 5:**
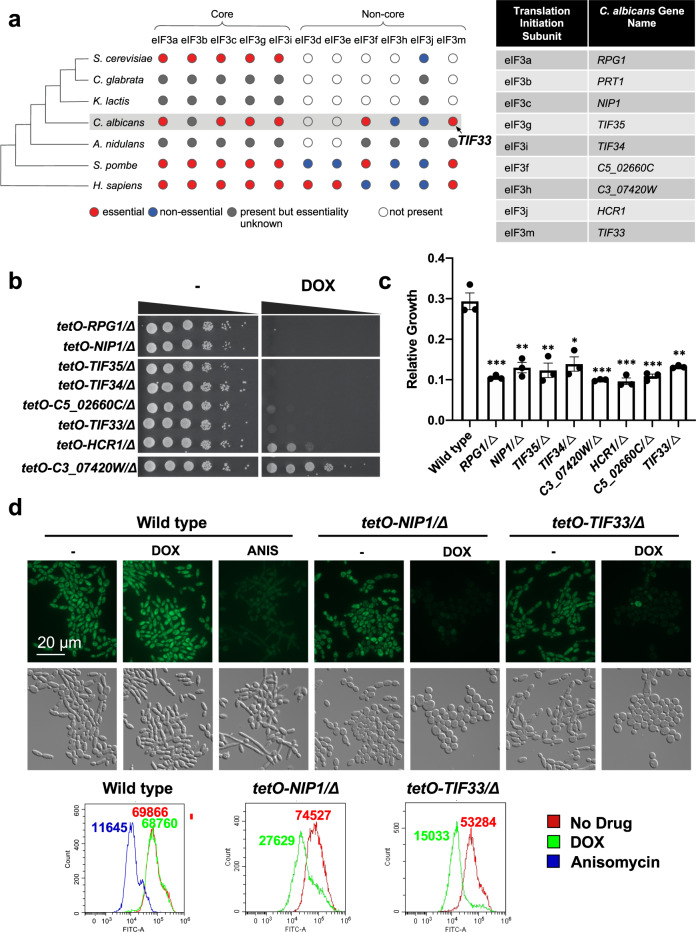
Characterization of Tif33 as a member of the translation initiation complex. **a** Phylogenetic tree highlighting divergence of eIF3 subunits across species. The presence of orthologs in the phylogenetic tree was derived from Wapinski et al.^99^, except for the *H. sapiens* orthologs, which were directly identified by PomBase^100^. Nodes are colored based on essentiality in the indicated species. The essentiality of *C. albicans* eIF3 genes was determined by our experimental test results. The essentiality of genes in *S. cerevisiae* and *S. pombe* was retrieved from *Saccharomyces* Genome Database^101^ and PomBase^100^, respectively. An eIF3 gene in *H. sapiens* was defined as essential if its CERES dependency score from the DepMap 21Q1 release^102,103^ was lower than −1.0 for more than 60% of the 808 CRISPR screens. **b** Testing the essentiality of eIF3 components. Strains were grown overnight in the absence or presence of 0.05 μg/mL doxycycline (DOX) at which point they were spotted in tenfold dilution (starting from an OD_600_ of 0.5) onto YNB agar alone or supplemented with 50 μg/mL DOX. Plates were photographed after growth for 48 h at 30 °C. **c** Heterozygous deletion mutants were grown in YPD at 30 °C in the presence or absence of nourseothricin (NAT) (8 μg/mL). Growth was measured after 24 h by OD_600_. Average growth between technical quadruplicate wells for each strain in the presence of NAT is plotted relative to the growth of that strain in the absence of NAT. Data are presented as average values ± SD. Significance of difference was determined by two-way ANOVA, Bonferroni correction for multiple comparisons, ***P < 0.001; **P < 0.01; *P < 0.05. Absolute *P* values provided in Source Data file. **d** A Click-iT protein synthesis assay kit was used to visualize protein translation. Strains were grown overnight in the absence or presence of 0.05 μg/mL DOX as indicated. Strains were subcultured to an OD_600_ of 0.1 in the same DOX conditions as the overnight and grown at 30 °C for 4 h. Cells were treated for 10 m with 100 μg/mL of the translation inhibitor anisomycin (ANIS), as indicated. The l-homopropargylglycine (HPG) alkyne methionine analog was added, and then the cells were fixed. The azide fluorophore was added, and cells were imaged on the GFP channel to detect if translation had occurred. Cells were analyzed by flow cytometry. Histograms depict relative fluorescence intensity (FITC-A) of a minimum 20,000 events, values depict median fluorescence intensity (MFI). Experiment was performed in biological duplicate with similar results. Source data are provided as a Source Data file.

Subsequently, we turned to the essential gene *C2_04370W*, an essential gene that although previously unannotated possesses significant sequence similarity to many genes encoding eukaryotic translation initiation factor 3 (eIF3) subunits. While the eIF3 complex is conserved in eukaryotes, the number of conserved subunits, as well as the essentiality of subunits, varies substantially across species (Fig. 5a). For example, the *S. cerevisiae* eIF3 complex contains a core complex of five essential subunits (eIF3a, eIF3b, eIF3c, eIF3g, and eIF3i) and a non-essential subunit (eIF3j)^47^. In contrast, the fission yeast *S. pombe*, encodes five core eIF3 subunits, and five non-core subunits (eIF3d, eIF3e, eIF3f, eIF3h, and eIF3m)^48^. While some eIF3 complex members have been predicted, the complex has yet to be investigated in *C. albicans*.

To identify all *C. albicans* eIF3 complex members, BLASTp was performed against the *C. albicans* genome with the *S. cerevisiae* and *S. pombe* subunits, identifying nine putative *C. albicans* eIF3 complex members. Six genes were predicted to function as members of the core complex: eIF3a (*RPG1)*, eIF3b (*PRT1*), eIF3c (*NIP1*), eIF3g (*TIF35*), and eIF3i (*TIF34*), while the non-core eIF3 complex was predicted to consist of eIF3h (*C3_07420W*), eIF3j (*HCR1)*, eIF3f (*C5_02660C*), and eIF3m (our gene of interest *C2_04370W*). In *S. pombe* the eIF3m subunit is encoded by *TIF313*, and therefore we renamed our gene of interest *TIF33* (translation initiation factor 33). Transcriptional repression of *RPG1*, *NIP1*, *TIF35*, *TIF34*, *C5_02660C*, or *TIF33* abolished growth, confirming their essentiality (Fig. 5b and Supplementary Fig. 3d). In contrast, *HCR1* and *C3_07420W* were deemed to be non-essential. The essentiality of *PRT1* could not be assessed due to insufficient transcriptional repression of the GRACE strain. These findings are consistent with observations that subunits associated with the core complex are highly conserved across eukaryotes and, wherever tested for viability, found to be essential, while the non-core members of the complex vary substantially both in terms of their presence and essentiality across species (Fig. 5a).

Next, we assessed if the putative eIF3 genes play a role in translation. We tested the sensitivity of heterozygous mutants to the translation inhibitor nourseothricin (NAT). This is based on the principle that modifying the expression of a compound target often alters the amount of compound required to inhibit that target^11,49^. Drug susceptibility analysis demonstrated that heterozygous mutants for all predicted eIF3 subunits were hypersensitive to NAT (Fig. 5c). Next, we performed a fluorescence-based translation assay. During active protein synthesis, an amino acid analog of methionine containing an alkyne moiety is incorporated into proteins. Subsequent click reaction between a green fluorescent azide and the alkyne allows for the qualitative detection of newly synthesized proteins using fluorescence microscopy and quantification by flow cytometry^50^. Treatment of a *C. albicans* wild-type strain with the translation inhibitor anisomycin caused a significant decrease in translation, as measured by a reduction in cellular fluorescence (Fig. 5d). Depletion of all putative translation initiation subunits, including the core eIF3c subunit *NIP1* and our uncharacterized gene *TIF33*, also caused a significant reduction in fluorescence (Fig. 5d and Supplementary Fig. 4a), indicating translation was impeded. This was specific to genes with roles in translation, as transcriptional repression of a control gene that was not involved in translation but was important for proliferation (*tetO-HSP90/hsp90Δ*) still resulted in strong fluorescence (Supplementary Fig. 4b). These results conclude Tif33 is an essential protein and a potential member of the eIF3 complex in *C. albicans*.

### Identification of an antifungal compound that targets *C. albicans* glutaminyl-tRNA synthetase

**Fig. 6:**
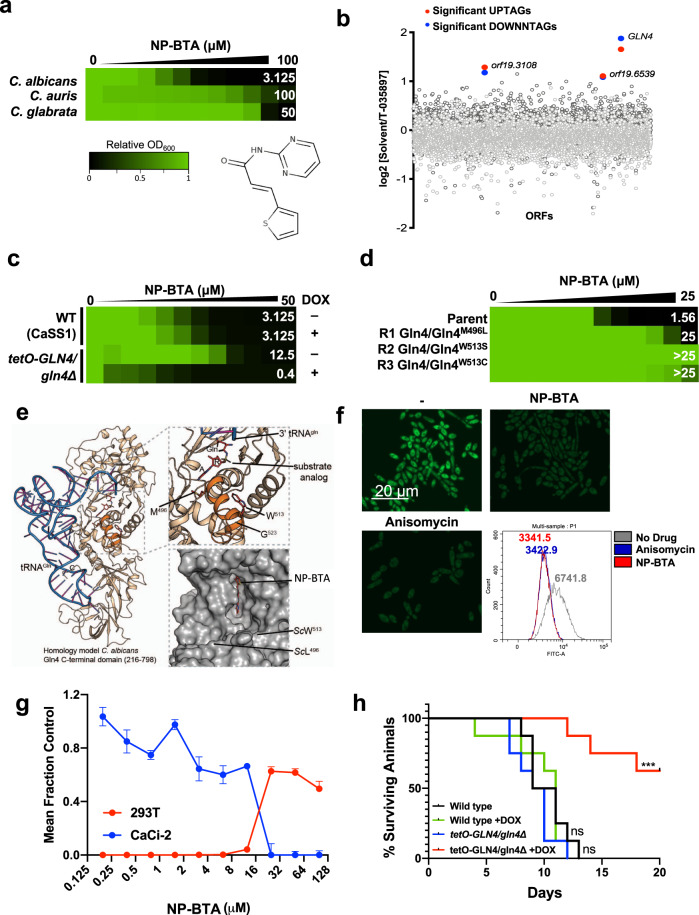
NP-BTA targets *C. albicans* glutaminyl-tRNA synthetase. **a** Dose−response assay based on twofold serial dilution of NP-BTA for *C. albicans* (SN95), *C. auris* (VPCI 673), or *C. glabrata* (F27). Assays were incubated for 24 h at 30 °C in YPD and growth was normalized relative to the respective no-compound control (see color bar). MIC_80_ values listed in white. Structure of NP-BTA displayed below heat map. **b** The double-barcoded *C. albicans* heterozygous deletion collection was grown in the presence or absence of NP-BTA (0.8 μM). Strains with a solvent/drug log2 ratio greater than 7 median absolute deviations (MADs) above the median were considered significant (see legend). UPTAG reads are shown in light gray, DOWNTAG reads are shown in dark gray. **c** Dose−response assay based on twofold serial dilution of NP-BTA for *C. albicans* (CaSS1) or *tetO-GLN4/gln4Δ* in the absence or presence of 0.05 μg/mL of doxycycline (DOX) as indicated. Assay performed as in (**a**). **d** Dose−response assay based on twofold serial dilution of NP-BTA for *C. albicans* parent (CaLC2749), as well as three independent resistant lineages (R1−R3). Identified Gln4 substitutions are listed. Dose−response assays were performed as in (**a**). **e** Homology model of the C-terminal domain of *C. albicans* Gln4 (beige, cartoon) based on the apo crystal structure of *S. cerevisiae* Gln4 (PDB: 4H3S; 66% sequence identity). To illustrate the location of the active site, tRNA^Gln^ (blue, cartoon) and glutaminyl aminoacyl-adenylate analog 5ʹ-O-[N-(L-glutaminyl)sulphamoyl]adenosine (red spheres; A: adenosine; R: ribose; Gln: glutamine) were placed from the *Escherichia coli* GlnRS-tRNA-substrate analog complex (PDB: 1QTQ), which aligned to the *C. albicans* model with an RMSD of 2.2 Å. Amino acids whose substitution confers reduced sensitivity to NP-BTA are shown as red sticks; numbering reflects amino acid position in *C. albicans* Gln4. Lower inset: Two binding poses of NP-BTA (sticks) were identified in the Gln4 active site after computational docking to the apo structure of *S. cerevisiae* Gln4 (gray surface). **f** Protein translation was evaluated as in Fig. 5. Cells were treated for 10 m with 100 μg/mL of anisomycin or 6.25 μM NP-BTA. Histograms depict relative fluorescence intensity (FITC-A) of events, values depict median fluorescence intensity (MFI). Experiment was performed in biological duplicate with similar results. **g** Relative growth/survival of human kidney-derived cells (HEK293T-luciferase, blue line) and azole-tolerant *C. albicans* in co-culture (CaCi-2-GFP, CaLC867, red line). Each point depicts the mean of triplicate wells. Error bars, SEM. Four-parameter curve fitting was performed in Prism v8.4. **h** Depletion of *GLN4* by the addition of DOX to the drinking water significantly improved survival relative to other conditions. Log-rank (Mantel-Cox) test, ***P < 0.0001. Log-rank test for trend, *P* = 0.0003. Source data are provided as a Source Data file.

Finally, in order to investigate the therapeutic potential of compounds targeting essential genes we surveyed data from a chemical screen we performed with 9600 compounds from the University of Tokyo’s Core Library that had been evaluated for single-agent activity against *C. albicans*. We prioritized one compound, N-pyrimidinyl-beta-thiophenylacrylamide (NP-BTA), for several reasons. First, it displayed potent antifungal activity against *C. albicans* with an MIC_80_ of 3.125 μM (Fig. 6a). Secondly, it exhibited moderate activity against the drug-resistant pathogens *Candida auris* and *Candida glabrata* (Fig. 6a). Finally, publicly available chemogenomic datasets^51^ suggested that NP-BTA targets glutaminyl-tRNA synthetase (*GLN4*) in *S. cerevisiae*, which we identified and confirmed as an essential gene in *C. albicans* (Supplementary Data 1 and 2).

To corroborate the *S. cerevisiae* results, haploinsufficiency (HIP) profiling was performed in *C. albicans*. The *C. albicans* double-barcoded heterozygous deletion library^52^ was pooled and cultured in the presence of 0.8 μM NP-BTA, a concentration that inhibited growth of the pool by ~20% relative to solvent-treated controls. Following high-throughput sequencing of strain-specific molecular barcodes, significantly hypersensitive hits were identified, revealing *GLN4* as the heterozygous mutant with the most significant barcode reduction upon NP-BTA treatment (Fig. 6b). Hypersensitivity of this mutant to NP-BTA was confirmed relative to the heterozygous deletion collection parental strain CaSS1 (Supplementary Fig. 5a). We further assessed the impact of overexpression of *GLN4* on NP-BTA sensitivity using the *tetO-GLN4/gln4Δ* mutant. In the absence of DOX we observed a ~3-fold increase in expression of *GLN4* relative to wild type (Supplementary Fig. 5b), which resulted in a four-fold increase in the NP-BTA MIC_80_ (Fig. 6c). As expected, DOX-mediated repression of *GLN4* sensitized *C. albicans* to NP-BTA (Fig. 6c).

As a complementary approach to decipher the target of NP-BTA, we performed selection experiments to identify *C. albicans* mutants with reduced susceptibility to the compound. A *C. albicans* drug-sensitized strain was employed that contains homozygous deletions in *CDR1, CDR2, MDR1*, and *FLU1* to enable the use of lower concentrations of compound for the selections and to reduce the likelihood of mutations that merely increase efflux. We isolated three independently evolved lineages with elevated resistance to NP-BTA that did not exhibit cross-resistance to fluconazole, a compound sensitive to enhanced efflux activity (Fig. 6d and Supplementary Fig. 5c). Sanger sequencing of the putative target in these strains revealed nonsynonymous mutations in *GLN4* (Fig. 6d), that mapped to the helical subdomain of the protein based on a homology model of *C. albicans* Gln4 that was generated based on the crystal structure of *S. cerevisiae* Gln4 (Fig. 6e)^53^. The interaction between an acceptor tRNA and the helical subdomain cause conformational changes in the active site of Gln4 that permit ATP/glutamine binding^54^. Computational docking of NP-BTA to *S. cerevisiae* Gln4 revealed multiple poses in which NP-BTA may bind to Gln4. The best ranked confirmation (IFD score: −24,709.48 kcal/mol) suggested that NP-BTA binds in the ATP/glutamine binding pocket (Fig. 6e). To further support that NP-BTA functions as a tRNA synthetase inhibitor, we assessed translation during NP-BTA treatment using the fluorescence-based translation assay. In comparison to untreated *C. albicans* cells, there was a significant reduction in fluorescence from cells treated with NP-BTA and the control translation inhibitor anisomycin (Fig. 6f), but not the control compound fluconazole, which inhibits growth but does not target translation (Supplementary Fig. 5d), strongly supporting NP-BTA as an inhibitor of Gln4.

To investigate the therapeutic potential of NP-BTA, we evaluated the efficacy of this molecule in a co-culture model, which enables assessment of both human and fungal cell proliferation in tandem^55^. Specifically, we used luciferase-tagged human HEK293T cells and a GFP-marked, fluconazole-tolerant *C. albicans* strain. Under tissue culture conditions, NP-BTA blocked *C. albicans* growth at 25 μM (Fig. 6g). Importantly, at this concentration NP-BTA simultaneously rescued human cell survival to a comparable degree as what was observed with fluconazole (Fig. 6g and Supplementary Fig. 5e). Motivated by the promising results in culture, we evaluated NP-BTA for susceptibility to hepatic inactivation by assessing its bioactivity after incubation in mouse liver microsome preparations. Technical controls included gepinacin, a compound sensitive to microsome-mediated inactivation, and fluconazole, a stable antifungal^56^. Although fluconazole maintained antifungal activity after incubation with mouse liver microsomes for 1 h at 37 °C with 5% CO_2_, both gepinacin and NP-BTA lost all activity against *C. albicans* (Supplementary Fig. 5f). This precluded testing the efficacy of this compound in an in vivo model of systemic candidiasis. However, as a proof-of-principle we tested the importance of Gln4 on *C. albicans* virulence. Mice were inoculated with either wild-type or *tetO-GLN4/gln4Δ* strains by retroorbital injection and supplied with drinking water supplemented with solvent or DOX to regulate the expression of *GLN4* in vivo (Fig. 6h). Only those mice infected with *tetO-GLN4/gln4Δ* that were provided with DOX in the drinking water saw improved survival relative to the other treatment groups, confirming *GLN4* is essential for *C. albicans* virulence in vivo (Fig. 6h, *p* < 0.001). For the three mice in the *tetO-GLN4/gln4Δ* with DOX treatment group that did succumb to the infection, this may have been due to insufficient transcriptional repression of *GLN4* in vivo, the emergence of suppressor mutations in particular strains that rendered the strain resistant to DOX treatment, or other factors. Thus, our work highlights the therapeutic potential of selectively targeting the essential protein Gln4 as an antifungal strategy.

## Discussion

Defining genes required for viability in pathogenic fungi not only unveils fundamental biological insights into the cellular processes that govern survival, but also reveals promising targets for therapeutic intervention. Unfortunately, genome-scale analyses of essentiality have primarily been conducted in *S. cerevisiae* with limited studies performed directly in fungal pathogens^18,20,57^. Here, we developed a machine-learning model to generate the most comprehensive essentiality prediction database for a fungal pathogen to date. To test the accuracy of our predictions, we dramatically expanded coverage of the *C. albicans* GRACE collection by 866 strains, and experimentally confirmed 621 genes as essential under standard laboratory conditions.

We note there has been a previous effort to build a machine-learning-based model for predicting essentiality in *C. albicans*, based on data from a TnSeq screen^18^. There are two important differences in our approach. First, we used a different gene essentiality standard for training the machine-learning approach; specifically, we used the existing GRACE strains as our gold standard while the previous study used a standard derived from ortholog information. Second, we incorporated a broader set of gene features as input to the model. Specifically, Segal et al.^18^ used TnSeq features as input, which are indeed powerful indicators of essentiality, while we used a broader set of gene features including not only TnSeq data from Segal et al.^18^, but also gene expression, information from orthologs, and sequence conservation and characteristics. In detailed comparisons of our approach and the previous machine-learning model, we show that both of these differences contribute to substantial improvement in the generalizability and accuracy of predictions for gene essentiality. In particular, our updated machine-learning model provides more accurate predictions of essentiality for genes represented among the recently constructed mutants in the GRACEv2 collection (Supplementary Note 1 and Supplementary Table 1).

One notable caveat to the interpretation of essential and non-essential genes determined here is that there may be instances where the DOX-repression system results in incomplete repression of the target gene. For essential genes whose function can be fulfilled with very low expression, such cases could be falsely identified as non-essential. Based on previous observations in *S. cerevisiae* and *E. coli*, one would expect a higher rate of these cases among genes with a low baseline level of expression^58^. We did find a limited number of cases consistent with this, including the fact that our analysis deemed the lowly-expressed MIND complex member Mtw1 as non-essential, despite previous reports^39^. As a result, we note that the machine-learning approach used to guide strain construction has also been trained with any biases related to the experimental system as we have used the confirmed essential and non-essential GRACE strains as our training standard. This has guided us to genes for which we are likely to confirm essential using our DOX-repression system, but this potential bias should be kept in mind when interpreting predictions outside the context of our experimental system.

As an opportunistic pathogen, *C. albicans* colonizes anatomically diverse mucosal surfaces and it must be able to adapt to many different environmental niches to thrive. While defining the essential gene set under laboratory conditions provides a critical foundation, analyses of genes essential for growth in vivo will undoubtedly reveal additional vulnerabilities. Several *C. albicans* genes have already been identified that are integral for growth in a murine model of *Candida* infection^59^, as well as required for *C. albicans* commensalism and virulence^17,60^, many of which are distinct from what has been observed in vitro. Additional studies have highlighted this discordance, concluding that although select genes are essential across multiple conditions (pan-essential genes), many show variable phenotypes in distinct environmental conditions. For example, chemical genomic assays in *S. cerevisiae* suggested nearly the entire yeast genome (97%) is required for optimal growth in at least one of the many distinct environmental conditions and stresses assessed^61^. Equally intriguing is fact that genetic background can modify gene essentiality in bacterial and fungal species^62,63^. This motivates continued exploration of genes required for *C. albicans* survival across different conditions to expand the antifungal target space and illuminate functional relationships in the cell.

Our analysis of gene essentiality revealed several fungal-specific essential genes with no previously annotated function. One such gene, *C1_01070C* or *KRP1*, was predicted to localize to the MIND complex, where it regulates chromosome segregation and cell cycle progression with other kinetochore-related proteins. To our knowledge, *S. cerevisiae* does not encode a homolog of *KRP1*, revealing divergent aspects of *C. albicans* biology. By coupling phenotypic characterization with a biochemical analysis to define the Krp1 interactome, we confirmed Krp1 likely functions at the *C. albicans* kinetochore. We also provided comprehensive characterization of all DASH/Dam1 complex members in *C. albicans*. The regulation of microtubule polymerization and kinetochore assembly during cell division serve as fundamental biological functions across all eukaryotes. Although many kinetochore components are evolutionarily conserved, there are divergent regulators that perform more specialized roles^64^. For example, the DASH/Dam1 complex is completely absent from metazoans^64–66^. Furthermore, although all complex members are essential in *S. cerevisiae*, they are not essential in *S. pombe*^34–37^. This species-specific essentiality has been attributed to differences in centromere organization and alternate kinetochore proteins^38^.

To further define the function of fungal-specific essential genes that lack a *S. cerevisiae* homolog, we next turned to *EMF1*. Leveraging co-expression datasets^22^, we predicted it to have a role in the mitochondria. Our discovery that Emf1 localizes to the *C. albicans* mitochondria and is important for maintaining mitochondrial integrity illustrates the power of co-expression partners in predicting gene function, a concept that has been employed for other species^67–70^. Furthermore, since targeting mitochondrial proteins has emerged as a promising therapeutic approach^59,71,72^, characterization of this mitochondrial gene in *C. albicans* provides insights relevant to fundamental biology with practical applications.

The essential genes identified in this study serve as a valuable resource to expand the antifungal drug target space. By integrating a comprehensive essentiality dataset with publicly available chemogenomic databases^51^, we highlight the potential of targeting the glutaminyl-tRNA synthetase Gln4 to combat *C. albicans* infections. While aminoacyl-tRNA-synthetases are essential for all organisms to attach amino acids to their cognate tRNAs for protein synthesis, we identified NP-BTA as a small molecule predicted to selectively target fungal Gln4 as it did not demonstrate significant mammalian toxicity in vitro. This is consistent with analyses demonstrating that many fungal aminoacyl-tRNA synthetases possess key structural differences from their mammalian homologs^73^. In addition, targeting aminoacyl-tRNA synthetases has been explored to treat infections caused by diverse bacteria and parasites, with the benzoxaborole compound tavaborole being the first aminoacyl-tRNA synthetase inhibitor approved for treatment of microbial infections^74,75^. As high-throughput sequencing and chemogenomic approaches continue to advance^11,76,77^, our ability to rapidly identify compounds predicted to target essential gene products will dramatically improve, catalyzing discovery and development of much-needed treatment options to combat life-threatening fungal disease.

## Methods

### Fungal strains and growth conditions

Strains used in this study are listed in Supplementary Table 2. Plasmids used in this study are listed in Supplementary Table 3. Archives of these strains were maintained in 25% glycerol at −80 °C. Active cultures were maintained on solid yeast extract peptone (YPD; 1% yeast extract, 2% bactopeptone, 2% glucose, and 2% agar) at 4 °C for no longer than 1 month. Liquid cultures were grown in YPD at 30 °C, unless otherwise indicated. To select for nourseothricin (NAT)-resistant mutants, NAT (Jena Bioscience, 96736-11-7) was solubilized in water and supplemented into YPD agar plates (150−250 μg/mL). Prototrophic colonies were selected on SD plates (0.674% yeast nitrogen base without amino acids with ammonium sulfate, 2% glucose, and 2% agar). Essentiality was tested on YNB plates (0.17% yeast nitrogen base without amino acids and without ammonium sulfate, with 2% glucose, 0.1% glutamic acid (MSG), and 2% agar).

### Building a machine-learning model for gene essentiality prediction

To identify essential and non-essential genes in *C. albicans*, we constructed a random forest (RF) classifier^25^ using the scikit-learn library^78^ (version 0.23.2) with the 13 features collected for 6638 *C. albicans* genes. We note that this gene set includes 6416 genes defined based on the A haplotype, 197 genes for which both A and B haplotype versions exist, and 5 genes that were unique to the B haplotype. In addition, there are 20 genes encoded on the mitochondrial genome. Details about the features used as input for our supervised machine-learning approach are in Supplementary Data 1. For genes with missing data for individual features, we imputed values using the mean of the corresponding attribute before training the RF classifier. Selected hyperparameters including the number of trees in the forest, the function to measure the quality of a split, the maximum depth of the tree, the number of features to consider for the best split, and the number of samples to draw from the training set, were optimized using fivefold cross-validation and a grid-search over a parameter grid. 80% of the 2327 genes in the *C. albicans* GRACE library were randomly selected for the training set, on which we conducted the fivefold cross-validation with grid-search. The performance on the remaining 20% of the GRACE gene set using the optimal classifier derived from the 80% GRACE training set is shown in Supplementary Fig. 2b.

### Experimental evaluation of the performance of model predictions

A set of 866 recently generated GRACE strains with a range of RF prediction scores were constructed for experimental validation. RF prediction scores for these genes were derived from a model trained on the complete GRACE gene set (all 2327 genes) with the previously determined optimal hyperparameters from the cross-validation described above. Prediction performance on this set was evaluated by performing receiver operating characteristic (ROC) and precision-recall analysis on the RF scores with respect to the experimentally determined essentiality verdict (see details in the “Methods” section “Testing for essentiality”). These predictions achieved an AP of 0.66 and AUC of 0.95 on this experimental validation set (Fig. 2b and Supplementary Fig. 2c). Permutation feature importance, which is defined to be the decrease in a model score when a single feature value is randomly permuted^25^, was evaluated on the GRACE gene set with 30 repeats and reported by scikit-learn.

### Mutant construction

Double-barcoded heterozygous (HET) mutants that were not available in the original collection were constructed, as described previously with some modifications^19,52^. The *CaHIS3* cassette was PCR amplified from pLC1251 using primers containing 43 base pairs of homology to the 5′ or 3′ of the target gene, internal unique 20 base pair strain-identifying barcodes with flanking common primer sequences, and 18 base pairs which anneal to the 5′ or 3′ of the *CaHIS3* gene (designated primer 5 and primer 6). The cassette was transformed into the parental strain for the library (CaLC6106), which contained the tetracycline-repressible transactivator and was auxotrophic for histidine. Prototrophic colonies were PCR tested for upstream and downstream integration using primer 3 or primer 7 together with a primer that anneals within the *HIS3* cassette (oLC6701 for primer 3 or oLC8017 for primer 7). Primer sequences used in this study are provided in Supplementary Data 7.

GRACE strains were generated from HET mutants, as described previously with some modifications^19^. Briefly, the NAT cassette and *tetO* promoter were PCR amplified from pLC763 using primers containing 20−22 base pairs complementary to the promoter replacement cassette and 70 base pairs of homology upstream or downstream of the start of the gene of interest (designated primer 1 and primer 2). The cassette was transformed into the relevant heterozygous mutant. NAT-resistant colonies were PCR tested for upstream integration of the *tetO* promoter using a primer that anneals ~250−350 base pairs upstream of the gene of interest (designated primer 3) and a primer that anneals within the NAT cassette (oLC6853), and/or downstream integration using a primer that anneals ~250−300 base pairs downstream of the start codon (designated primer 4) and a primer that anneals within the *tetO* cassette (oLC2535). NAT-resistant colonies were additionally tested by PCR to verify the absence of a wild-type promoter upstream of a wild-type allele of the target gene using primer 3 and primer 4.

#### CaLC7321

This strain with both copies of *C6_03200W* or *EMF1* C-terminally tagged with GFP was made using a transient CRISPR approach adapted from Min et al.^79^. The *GFP-NAT* cassette was PCR amplified from pLC389 using oLC9284 and oLC9285. The *CaCAS9* cassette was amplified from pLC963 using oLC6924 and oLC6925. The sgRNA fusion cassette was PCR amplified from pLC963 with oLC5978 and oLC9288 (fragment A) and oLC5980 and oLC9289 (fragment B), and fusion PCR was performed on fragments A and B using the nested primers oLC5979 and oLC5981. The *GFP-NAT* cassette, sgRNA, and Cas9 DNA were transformed into CaLC239. Upstream integration was PCR tested using oLC600 and oLC9286, and downstream integration was tested using oLC274 and oLC9287. Lack of a wild-type allele was PCR tested using oLC9290 and oLC9286.

#### CaLC7334

This strain with both copies of *C1_01070C* or *KRP1* C-terminally tagged with GFP was made using a transient CRISPR approach adapted from Min et al.^79^. The *GFP-NAT* cassette was PCR amplified from pLC389 using oLC9334 and oLC9335. The *CaCAS9* cassette was amplified from pLC963 using oLC6924 and oLC6925. The sgRNA fusion cassette was PCR amplified from pLC963 with oLC5978 and oLC9338 (fragment A) and oLC5980 and oLC9339 (fragment B), and fusion PCR was performed on fragments A and B using the nested primers oLC5979 and oLC5981. The *GFP-NAT* cassette, sgRNA, and Cas9 DNA were transformed into CaLC239. Upstream integration was PCR tested using oLC600 and oLC9336, and downstream integration was tested using oLC274 and oLC9337. Lack of a wild-type allele was PCR tested using oLC9337 and oLC9340.

#### CaLC7408

This strain with both copies of *C6_03200W* or *EMF1* C-terminally tagged with GFP and both copies and *GCF1* C-terminally tagged with RFP was made using a transient CRISPR approach adapted from Min et al.^79^. The *RFP-ARG* cassette was PCR amplified from pLC1208 using oLC9381 and oLC9382. The *CaCAS9* cassette was amplified from pLC963 using oLC6924 and oLC6925. The sgRNA fusion cassette was PCR amplified from pLC963 with oLC5978 and oLC9385 (fragment A) and oLC5980 and oLC9386 (fragment B), and fusion PCR was performed on fragments A and B using the nested primers oLC5979 and oLC5981. The *RFP-ARG* cassette, sgRNA, and Cas9 DNA were transformed into CaL7321. Upstream integration was PCR tested using oLC6971 and oLC9383, and downstream integration was tested using oLC6970 and oLC9384. Lack of a wild-type allele was PCR tested using oLC9387 and oLC9384.

#### CaLC7410

This strain with both copies of *C1_01070C* or *KRP1* C-terminally tagged with GFP and both copies and *MTW1* C-terminally tagged with RFP was made using a transient CRISPR approach adapted from Min et al.^79^. The *RFP-ARG* cassette was PCR amplified from pLC1208 using oLC9374 and oLC9375. The *CaCAS9* cassette was amplified from pLC963 using oLC6924 and oLC6925. The sgRNA fusion cassette was PCR amplified from pLC963 with oLC5978 and oLC9378 (fragment A) and oLC5980 and oLC9379 (fragment B), and fusion PCR was performed on fragments A and B using the nested primers oLC5979 and oLC5981. The *RFP-ARG* cassette, sgRNA, and Cas9 DNA were transformed into CaLC7334. Upstream integration was PCR tested using oLC6971 and oLC9376, and downstream integration was tested using oLC6970 and oLC9377. Lack of a wild-type allele was PCR tested using oLC9380 and oLC9377.

#### CaLC7411

This strain with both copies of *C1_01070C* or *KRP1* C-terminally tagged with GFP and both copies and *DAD1* C-terminally tagged with RFP was made using a transient CRISPR approach adapted from Min et al.^79^. The *RFP-ARG* cassette was PCR amplified from pLC1208 using oLC9388 and oLC9389. The *CaCAS9* cassette was amplified from pLC963 using oLC6924 and oLC6925. The sgRNA fusion cassette was PCR amplified from pLC963 with oLC5978 and oLC9392 (fragment A) and oLC5980 and oLC9393 (fragment B), and fusion PCR was performed on fragments A and B using the nested primers oLC5979 and oLC5981. The *RFP-ARG* cassette, sgRNA, and Cas9 DNA were transformed into CaLC7334. Upstream integration was PCR tested using oLC6971 and oLC9390, and downstream integration was tested using oLC6970 and oLC9391. Lack of a wild-type allele was PCR tested using oLC9394 and oLC9391.

### Plasmid construction

Cloning procedures were performed following standard protocols. Plasmids used in this study are listed in Supplementary Table 3.

#### pLC1251

This vector is used to efficiently generate heterozygous deletion mutants using an analogous approach as to what was used to generate the original double-barcoded heterozygous deletion collection (HET)^52^. Ca*HIS3* was amplified from genomic DNA from DBC strain 181 (*orf19.1702*), using primers oLC7860 and oLC7861 (which bind to priming sites U2 and D2). This amplicon and pLC1237 (pUC19) were digested with *Bam*HI overnight, ligated, and transformed into DH5alpha. A diagnostic digest with *Bam*HI was performed. Sequencing was performed with common primers M13 Forward and M13 Reverse and oLC6701.

#### pLC1208

The RFP coding sequence was amplified from pLC447 using oLC7964 and oLC7965. The PCR amplicon was digested with *Bam*HI and *Asc*I and inserted into pLC1100 (pFA-3HA-ARG) between the same sites.

### Testing for essentiality

*C. albicans* GRACE strains were pinned into 96-well plates (Sarstedt) containing liquid YPD medium and grown overnight at 30 °C. The next day, cells were transferred into liquid YPD medium in the absence and presence of 100 µg/mL DOX and grown at 30 °C overnight. Approximately 2 μL of cultures were then transferred onto YNB agar plates in the absence and presence of 100 µg/mL DOX using a metal replicator and grown for 2 days at 30 °C. The plates were imaged and essentiality was scored by two independent people, using the scoring system outlined in Supplementary Fig. 1a. Genes were scored as essential only if the mutant had a severe growth defect in the presence of DOX and grew robustly in the absence of DOX. One biological replicate was performed for the original GRACE strains and essential verdicts were compared with a previous essentiality screen of the same library^20^. Two biological replicates were performed for the recently constructed GRACEv2 strains. Essentiality verdicts that were not consistent were retested and rescored through spot dilution assays. For spot dilution assays, strains were diluted to an OD_600_ equal to 0.5 and tenfold cell dilutions were performed. 3 μL of diluted inoculum was plated onto YNB agar medium in the absence or presence of 50 μg/mL DOX. Plates were imaged after 48 h at 30 °C.

### Data processing

To identify the *S. cerevisiae* orthologs of *C. albicans* essential genes, *S. cerevisiae* ortholog data by *Candida* Gene Order Browser (CGOB) was downloaded from the *Candida* Genome Database (CGD) website (http://www.candidagenome.org/download/homology/orthologs/C_albicans_SC5314_S_cerevisiae_by_CGOB/)^21^. For genes without predicted orthologs in *S. cerevisiae*, the best hits from other species were explored using data from BLASTp analysis available on the CGD website (http://www.candidagenome.org/download/homology/best_hits/C_albicans_SC5314_S_cerevisiae_best_hits.txt). Gene Ontology (GO) was identified using the CGD Gene Ontology finder. Conserved Domain Searches were performed using the NCBI Conserved Domain Search (https://www.ncbi.nlm.nih.gov/Structure/cdd/wrpsb.cgi)^80^. Protein Basic Local Alignment Search Tool (BLAST) searches were performed on the NCBI website (https://blast.ncbi.nlm.nih.gov/Blast.cgi?PAGE = Proteins)^81,82^, on the FungiDB website (fungidb.org)^83^, and on the CGD website.

### Identification of fungal-specific genes without human orthologs

To identify fungal-specific *C. albicans* genes among our 621 validated essential genes, we utilized data from the public orthology databases MetaPhOrs^84^ (version 2.0) and InParanoid^85^ (version 8.0). Orthologs were identified using the default recommended cutoffs of 0.5 for Orthology Consistency Score and 1 for Evidence Level in MetaPhOrs, as well as with bootstrap support in InParanoid.

### BLASTp analysis to assign gene function

All DASH/Dam1 complex members were previously identified^21,65^ and their identity was confirmed with BLASTp analysis. For MIND complex members, genes were annotated based on their *S. cerevisiae* homologs: *NSL1* (*C7_01830W*), *MTW1* (*C2_09840W*), and *NNF1* (*CR_01360W*). No *C. albicans* ortholog could be identified for *DNS1*. To identify all the *C. albicans* eIF3 complex members, BLASTp searches were performed against the *S. cerevisiae* and *S. pombe* subunits, identifying 11 putative *C. albicans* eIF3 complex members. Six genes were predicted to be associated with the core complex: eIF3a (*RPG1)*, eIF3b (*PRT1*), eIF3c (*NIP1*), eIF3g (*TIF35*), and eIF3i (*TIF34*). The non-core eIF3 complex was predicted to consist of eIF3h (*C3_07420W*), eIF3j (*HCR1)*, eIF3f (*C5_02660C*) and eIF3m (our gene of interest *C2_04370W* or *TIF33*).

### Co-expression UMAP cluster analysis

853 bulk RNAseq runs from 18 large-scale studies of *C. albicans* available through the NCBI sequence read archive were aggregated^22^. Gene Log(FPKM + 1) expression vectors were reduced to two dimensions and clustered using Monocle3^86^ by pre-processing with PCA to 500 dimensions, reducing dimensions using the UMAP algorithm^87^ with the following non-default parameters (*a* = 50, *b* = 0.5, n_neighbors = 30, negative_sample_rate = 50, repulsion_strength = 3, n_epochs = 2000, and metric = ‘cosine’), and then clustered using the Leiden network modularity algorithm^88^ with non-default parameters (*k* = 30, resolution 1e−5, num_iter = 10). The genes in each cluster were run through the CGD GO term enrichment tool to identify potential gene function. Gene UMAP coordinates, gene clusters, and the leading gene-function signature were plotted using ggplot2 ^89^.

### Morphogenesis assays

Overnight cultures of the indicated strains were grown in YPD at 30 °C, shaking, and subcultured to an OD_600_ of 0.1 in YPD in the absence and presence of 0.1 µg/mL DOX. Cultures were grown at 30 °C overnight before imaging on a Zeiss Axio Imager.MI.

### Reverse transcriptase quantitative PCR (RT-qPCR)

YPD overnights of the wild-type strain and the GRACE mutants were subcultured to an OD_600_ of 0.1 in YPD in the absence and presence of 0.05 µg/mL DOX for mitochondrial-related genes, the putative eIF3 subunit and *GLN4* GRACE strains, and 0.1 µg/mL DOX for the Dam1/DASH and MIND complex GRACE strains. Cultures were grown overnight at 30 °C, shaking. The next day, the cultures were subcultured again to an OD_600_ of 0.1 in YPD in the absence and presence of the same concentration as above. Cultures were grown at 30 °C, shaking for ~4 h or until in mid-log phase. Cells were pelleted, washed once with cold 1× phosphate buffered saline (PBS), flash-frozen, and stored at −80 °C. Cells were lysed by bead beating four times for 30 s, with 1 min on ice between. RNA was extracted using the QIAGEN RNeasy kit and DNase treated using the QIAGEN RNase free DNase Set. cDNA synthesis was preformed using the iScript cDNA synthesis Kit (BioRad). qRT-PCR was performed in technical triplicate with a 10 µL reaction volume in a 384-well plate, using Fast SYBR Green Master Mix (Applied Biosystems) and the BioRad CFX-384 Real Time System. The following cycling conditions were used: 95 °C for 3 min, then 95 °C for 10 s and 60 °C for 30 s, for 40 cycles. The melt curve was completed with the following cycle conditions: 95 °C for 10 s and 65 °C for 10 s with an increase of 0.5 °C per cycle up to 95 °C. The primers used to monitor expression are listed in Supplementary Data 7.

### Fluorescence microscopy

Overnights of wild-type, *tetO-TIM12/tim12Δ*, and *tetO-EMF1/emf1Δ* strains were grown in YPD in the absence and presence of 0.05 µg/mL DOX at 30 °C with shaking. Cells were subcultured to an OD_600_ of 0.1 in YPD in the same DOX conditions and grown for 3 h at 30 °C with shaking. Cultures were further incubated with 50 nM Mitotracker Red for 40 min, then washed and resuspended in PBS. Fluorescent images with the DsRed channel (Mitotracker Red) were taken on a Zeiss Axio Observer.Z1 with Zeiss ApoTome Attachment, with exposure time remaining constant among samples.

For co-localization experiments, overnights for strains CaLC7321 (*EMF1*-GFP/*EMF1*-GFP), CaLC7408 (*EMF1*-GFP/*EMF1*-GFP *GCF1*-RFP/*GCF1*-RFP), CaLC7410 (*KRP1*-GFP/*KRP1*-GFP *MTW1*-RFP/*MTW1*-RFP, CaLC7411 (*KRP1*-GFP/*KRP1*-GFP *DAD1*-RFP/*DAD1*-RFP) were grown in YPD at 30 °C with shaking. All strains were subcultured to an OD_600_ of 0.1 in YPD and grown for 4 h at 30 °C with shaking. The CaLC7321 subculture was treated with Mitotracker Red as above. The CaLC7408 subculture was washed and resuspended in PBS, then incubated with 1 µg/mL 4′,6-diamidino-2-phenylindole (DAPI) for 1 h on a rotating platform at room temperature. The CaLC7410 and CaLC7411 subcultures were washed and resuspended in PBS then incubated with 5 µg/mL Hoechst 33342 for 10 min on a rotating platform at room temperature. Cells were imaged by differential interference (DIC) microscopy and using the GFP channel (Emf1-GFP and Krp1-GFP), DsRed channel (Mitotracker Red, Dad1-RFP, Mtw1-RFP and Gcf1-RFP), and DAPI channel (DAPI and Hoechst 33342) on a Zeiss Axio Observer.Z1 microscope with Zeiss ApoTome attachment. For all ApoTome images, five phase images were captured, and global bleaching correction was applied.

### Quantifying relative *NAD2* copy number

Overnights of wild-type, *tetO-EMF1/emf1Δ*, and *tetO-GCF1/gcf1∆* strains were grown in YPD in the absence and presence of 0.05 µg/mL DOX at 30 °C with shaking. Cells were subcultured to an OD_600_ of 0.1 in YPD under the same DOX and growth conditions for 8 h before pelleting and storage at −80 °C. Total genomic DNA was extracted from cell pellets using the Invitrogen PureLink Genomic DNA Kit and quantified using the Invitrogen Quant-iT PicoGreen dsDNA Assay Kit. Total genomic DNA (20 ng) was analyzed by qPCR in technical triplicate with a 10 µL reaction volume in a 384-well plate, using Fast SYBR Green Master Mix (Applied Biosystems) and the BioRad CFX-384 Real Time System. The following cycling conditions were used: 95 °C for 3 min, then 95 °C for 10 s and 60 °C for 30 s, for 40 cycles. The melt curve was completed with the following cycle conditions: 95 °C for 10 s and 65 °C for 10 s with an increase of 0.5 °C per cycle up to 95 °C. The primers used to monitor expression are listed in Supplementary Data 7. Relative *NAD2* copy number was calculated by first normalizing *NAD2* levels with *ACT1* and *GPD1* levels for each respective sample and then normalized to wild type in the absence of DOX. Experiments were performed with two biological replicates. BioRad CFX Manager (version 3.1) was used to normalize RT-qPCR data and plot mean values with calculated SEM.

### Mass spectrometry

AP-MS experiments were performed as previously described^90,91^, with minor modifications. Briefly, the GFP-tagged Krp1 (C1_01070c) strain was grown overnight at 30 °C in YPD. Stationary phase cultures were diluted to an OD_600_ of 0.1 in 1 L YPD and grown to an OD_600_ between 0.6 and 0.8. Cells were harvested at 1720 × *g* for 30 min at 4 °C, washed with ice-cold water, and snap-frozen in an ethanol-dry ice bath. As a control, GFP-tagged Eno1 was prepared in the same manner. For protein extraction, samples were diluted 1:1 by weight in lysis buffer (50 mM HEPES [pH 7.5], 150 mM NaCl, 5 mM EDTA, 5 mM DTT, 0.1% NP-40, 1× ROCHE protease inhibitor cocktail tablet [Roche Diagnostics, Mississauga, ON, Canada]) and vortexed with glass beads (0.5 mm) for 4 × 1 min. Lysates were collected by stacked transfer for 1 min at 100 × *g* with a 27½-gauge needle and clarified by centrifugation at 16,110 × *g* for 20 m in 4 °C in a microcentrifuge.

To affinity purify the GFP-interacting proteins, we used the GFP trap affinity resin (ChromoTek, Martinsried, Germany). The resin was equilibrated three times with 1 mL of lysis buffer (50 mM HEPES [pH 7.5], 150 mM NaCl, 5 mM EDTA, 5 mM DTT, 0.1% NP-40, 1× ROCHE protease inhibitors [Roche Diagnostics, Mississauga, ON, Canada]), using 25 μL resin for each 1 L culture. The protein extract was then added to the resin and rotated for 2 h at 4 °C. The beads were then washed with 1 mL of lysis buffer and then 1 mL of wash buffer (20 mM Tris [pH 8.0], 2 mM CaCl_2_). The samples were digested on-bead with 0.75 μg trypsin (0.2 μg/μL in 20 mM Tris-HCl, pH 8.0) at 37 °C overnight with rotation. The beads were then magnetized, and the supernatant was transferred to a fresh tube and incubated with 0.5 μg trypsin without rotation at 37 °C for 4 h. Formic acid was added to a final concentration of 1–2% and the samples were kept at −80 °C until used for MS.

AP-MS samples and controls were analyzed by MS in two biological replicates, as previously described^90,91^, with the following modifications. Samples in 5% formic acid were directly loaded at 800 nl/min for 20 min onto a 100 µm × 15 cm nano-spray emitter. Peptides were eluted from the column with an acetonitrile gradient generated by an Eksigent ekspert™ nanoLC 425 and analyzed on a TripleTOF^™^ 6600 instrument (AB SCIEX, Concord, Ontario, Canada). The gradient was delivered at 400 nL/min from 2% acetonitrile with 0.1% formic acid to 35% acetonitrile with 0.1% formic acid using a linear gradient of 90 min. This was followed by a 15-min wash with 80% acetonitrile with 0.1% formic acid, and equilibration for another 15 min to 2% acetonitrile with 0.1% formic acid. The MS1 scan had an accumulation time of 250 ms within a mass range of 400−1800 Da. This was followed by ten MS/MS scans of the top ten peptides, with accumulation time of 100 ms for each MS/MS scan. Each candidate ion was required to have a charge state from 2 to 5 and a minimum threshold of 300 counts per second, isolated using a window of 50 mDa. Previously analyzed candidate ions were dynamically excluded for 7 s. MS data generated were stored, searched, and analyzed using the ProHits laboratory information management system (LIMS) platform^92^ and searched using Mascot (v2.3.02) and Comet (v2012.02 rev.0) against the *C*. *albicans* RefSeq database (version 68), as previously described^90,91^. The database parameters were set to search for tryptic cleavages, allowing up to two missed cleavage sites per peptide, with a mass tolerance of 35 ppm for precursors with charges of 2+ to 4+ and a tolerance of ±0.15 amu for fragment ions. Variable modifications were deamidated asparagine and glutamine residues and oxidized methionine residues. The results from each search engine were analyzed through the Trans-Proteomic Pipeline (TPP v4.6 OCCUPY rev 3)^93^ via the iProphet pipeline^94^. SAINTexpress (v3.3)^95^ was used as a statistical tool to calculate the probability value of each potential protein–protein interaction from background contaminants using default parameters and a ProteinProphet cutoff of 0.95 and a minimum of two unique peptides. Controls were kept uncompressed and a BFDR of 1% or lower was required for proteins to be classified as significant interaction partners. Cytoscape (v3.8.2) was used to generate Krp1 interaction network. Proteomics data were deposited in the ProteomeXchange database (PXD029002) through partner MassIVE (massive.ucds.edu; MSV000088204).

### Growth curve assays

Wild-type and heterozygous deletion mutants were grown in YPD at 30 °C overnight, shaking. Dose−response assays with NAT or NP-BTA were performed according to standard protocols^96^ in 96-well or 384-well, flat-bottom microtiter plates (Sarstedt). Growth at 30 °C was determined by measuring OD_600_ using a CG-12 Cell-Grower Robot, third generation (S&P Robotics) at 24 h (for NAT sensitivity) or monitored over every 15 min 24 h (for NP-BTA). Relative growth of the mutants for NAT sensitivity was calculated by normalizing the OD_600_ of each mutant in 8 µg/mL NAT compared to growth in no drug, and was plotted relative to the normalized growth of the wild-type strain. For NP-BTA sensitivity, growth of the mutants was determined by normalizing the ratio of the area under the curve (AUC) in the presence of drug to the AUC in drug-free medium against that of the wild-type control.

### Dose−response assays and cidality testing

Antifungal drug susceptibility was measured using a modified broth microdilution protocol in YPD medium and 96-well plate (Sarstedt) format, as previously described^96^. Following OD_600_ measurements, cultures were stamped onto compound-free YPD agar to assess cidality. GRACE strains were tested in YPD medium in the presence or absence of 0.05 µg/mL and 100 µg/mL DOX as indicated. Data were displayed quantitatively as heat maps using Java TreeView software (version 1.1.6r4). Reported results are representative of two independent experiments, each performed in technical duplicate.

### Translation assay

Translation assays were performed with the Click-iT® HPG Alexa Fluor® 488 Protein Synthesis Assay Kit (Thermo Fisher C10428), according to manufacturer’s instructions with some modifications. Overnights of wild-type and GRACE strains were subcultured in YPD in the presence and absence of 0.05 µg/mL DOX and grown at 30 °C overnight, shaking. Cells were subcultured to an OD_600_ of 0.2 into 10 mL synthetic defined (SD) medium without amino acids, without ammonium sulfate supplemented with 2% glucose, monosodium glutamate, histidine and uridine in the presence and absence of 0.05 µg/mL DOX and grown at 30 °C, shaking, to mid-log phase. For compound treatment, 2 mL aliquots of wild-type cells were spun down and the pellet was resuspended in SD with or without 100 µM anisomycin or 6.25 µM NP-BTA. Cultures were incubated with or without inhibitors for 10 min in shaking conditions at 30 °C, before adding the L-homopropargylglycine (HPG) alkyne methionine analog at a 1:1000 dilution. For the genetic depletion samples, equal amounts of cells were spun down from each of the samples and the supernatant was removed. Pellets were resuspended in 1 mL of SD in the presence and absence of 0.05 µg/mL DOX with a 1:1000 dilution of HPG. All HPG treated samples were incubated at 30 °C, shaking, for 15 min. Cells were spun down, supernatant removed, and pellets washed once with 1× PBS before resuspended in 500 µL PBS. Cells were fixed by adding 500 µL 70% ethanol in 1× PBS and rocking for 1 h at room temperature. Cells were then pelleted and washed twice with 3% BSA in PBS. Pellet was resuspended with 500 µL of the reaction cocktail containing the azide fluorophore (prepared as according to the manufacturer’s protocol) and incubated for 30 min, shaking in the dark. The samples were washed once with the rinse buffer, pelleted and resuspended in 75 µL of 1× PBS before imaging. Cells were imaged by differential interference contrast microscopy and the EGFP channel on a Zeiss Axio Imager.MI at the same exposure time. To quantify the fluorescence of each sample, a CytoFlex Flow Cytometer (Beckman Coulter) was used. A 1:10 dilution of the cell suspension was prepared in PBS in a flat-bottom, transparent, 96-well plate (Beckman Coulter) to a final volume of 200 µL. Each sample was run using the CytExpert Software (version 2.4) until ~20,000 events had been recorded. Appropriate gating strategies were applied to all samples (Supplementary Fig. 6). Histograms show the FITC value for each event in a population following gating to exclude debris and doublets.

### Haploinsufficiency profiling (HIP)

Double-barcoded, heterozygous (HET) deletion mutant pools stored in 25% glycerol at −80 °C were diluted to OD_600_ = 0.1 in YPD and grown for 90 min at 30 °C, shaking. Cultures were diluted twofold into a final volume of 5 mL YPD in the presence or absence of 0.8 µM NP-BTA (dissolved in DMSO) and grown in triplicate for 18 h at 30 °C, shaking. Cells were harvested by centrifugation and cell pellets were stored at −80 °C. Genomic DNA was extracted (PureLink Genomic DNA Mini Kit, Invitrogen) and barcodes were PCR amplified from 56 ng of genomic DNA. Universal and indexed UPTAG and DNTAG barcode primers are described in Supplementary Data 7. UPTAG and DNTAG barcode pools were combined in an equal ratio and sequenced on an Illumina NextSeq500 instrument (Mid-Output, V2 Chemistry). Sequencing primers are described in Supplementary Data 7. UPTAG and DNTAG read frequencies for each strain were compiled into separate indexed samples, representing technical triplicates for the solvent control and drug conditions. Strains whose UPTAGs or DNTAGs in the solvent control were <20% of the median read frequency were excluded from analysis. Log2 fold changes in the UPTAG and DNTAG representation for each strain were calculated. Strains were considered to be significantly reduced in abundance if the log2 fold-change in the solvent over drug condition was >7 median absolute deviations (MADs) above the overall median in both the UPTAG and DNTAG, or if either barcode was >7 MADs and the other barcode was omitted due to insufficient reads.

### Resistant mutation selection experiments

The *C. albicans* drug-sensitized background (CaLC2749) was used to evolve three independent lineages on solid YPD plates containing 6.24 µM NP-BTA. 1 × 10^8^ cells were plated and incubated at 30 °C for 3−5 days until single colonies appeared. PCR amplification of *GLN4* using primers oLC9302 and oLC9303, followed by Sanger sequencing of the PCR products using primers oLC9304-oLC9311 was performed to identify resistance-conferring mutations in *GLN4*.

### Homology modeling and computational docking

A homology model of *C. albicans* Gln4 was generated by one-to-one threading using the Phyre2 server^97^, using *S. cerevisiae* Gln4 as a template (PDB: 4H3S). Computational docking of NP-BTA was performed using Glide (Maestro release 2020-4; Schrödinger LLC, New York, NY, 2020)^98^. Using the Maestro Protein Preparation module, *S. cerevisiae* Gln4 was imported (PDB 43HS), waters were removed, hydrogens were added, Het states were generated (Epik; pH 7.5), H-bonds were assigned/optimized (PROPKA; pH 7.5), and restrained minimization was performed (Impref; heavy atoms restrained 0.3 Å). NP-BTA was imported using the LigPrep module; possible ionization states were generated (Epik; pH 7.5), and all possible tautomers/stereoisomers were generated. Docking of NP-BTA was performed using the Induced Fit Docking module, which performs rigid-body docking (Glide), followed by localized minimization (Prime), and re-docking (Glide). The resultant poses were ranked using the induced fit docking score.

### Co-culture assays

The HEK293T (CRL-3216) cell line was purchased from the American Type Culture Collection (ATCC) and maintained at 37 °C and 5% CO_2_ in DMEM medium (Sigma, D5796) supplemented with 10% fetal bovine serum (FBS, Gibco 16000044). Experiments were performed using cells within 15 passages post-recovery from low-passage stocks stored in liquid nitrogen, and confirmed by PCR to be negative for mycoplasma contamination. Human HEK293T cells expressing firefly luciferase were seeded at 2000 cells/well in a 384-well plate (Sarstedt) in DMEM supplemented with 10% FBS. After adherence overnight, GFP-labeled *C. albicans* CaCi-2 (CaLC867) was added at 25,000 cells/mL in an equal volume. Twofold dilutions of NP-BTA or fluconazole were then added to the wells and cells were incubated for 72 h. Relative fluorescence (480 nm excitation, 540 nm emission) per well was measured. 5 µL Steady-Glo Luciferase Assay (Promega Cat #E2520) reagent was then added per well and incubated for 15 min, followed by measurement of relative luminescence per well. Both fluorescence and luminescence readouts were obtained using a Tecan Spark microplate reader. Reported results are representative of two independent experiments, each performed in technical triplicate.

### Microsome stability assay

Microsome stability assay was performed as described previously^56^. In brief, 3 μM or 6 μM NP-BTA was incubated in mouse liver microsomes (0.5 mg/mL) and an NADPH-regenerating buffer system (Xenotech L1500), or in buffer alone. 50 μM fluconazole and 40 μM gepinacin were included as metabolically stable and unstable control compounds, respectively. Reaction mixtures were incubated for 1 h at 37 °C + 5% CO_2_. Microsomes were inactivated by addition of 1 mM PMSF and incubated for an additional 30 min. Reaction mixtures were then diluted 1:4 in YPD medium supplemented with 1× penicillin-streptomycin containing *C. albicans* (SN95) inoculum at OD_600_ = 0.0005. Cultures were incubated for 24 h at 30 °C and fungal growth was assessed by OD_600_ measurement. Reported data are representative of two independent experiments, each performed in technical quadruplicate.

### Mouse model of *Candida* infection

For the time to illness assay, groups of 8- to 10-week-old female BALB/c mice (Charles River) were received and treated with drinking water for 1 day (8 mice per group, 4 mice per cage). The next day, 16 mice were given drinking water containing 5% glucose with doxycycline hyclate (final concentration 250 μg/mL) (TGI:: 24390-14-5) for 7 days prior to the infection. Meanwhile, 16 mice received drinking water with 5% glucose only as a control. Glucose was included to ensure sufficient uptake of the water. Strains were grown in YEPD overnight at 30 °C. Cells were subsequently diluted to OD_600_ 0.1 in 100 mL YEPD broth and incubated at 30 °C for 4 h with constant shaking. Cells were collected and washed twice with 0.9% saline (Baxter:2F7123). The number of cells was counted by hemocytometer and 4 × 10^5^ cells were injected retro-orbitally into each mouse. The inoculum was confirmed by plating on a YPD agar plate and CFU counts after 2 days of growth. Mice were monitored daily and euthanized when was moribund (hunched posture, minimal motor activity, or BCS of 2 or less). Rodent housing room temperature ranges of 69−74 °F with 30−70% humidity. A 12 h light/12 h dark cycle was used. Animal experiments were conducted with approval from UCSF Institutional Animal Care and Use Committee (protocol number AN189431-01).

### Reporting summary

Further information on research design is available in the Nature Research Reporting Summary linked to this article.

## Supplementary information

Supplementary Information

Description of Additional Supplementary Files

Supplementary Data 1

Supplementary Data 2

Supplementary Data 3

Supplementary Data 4

Supplementary Data 5

Supplementary Data 6

Supplementary Data 7

Reporting Summary

## Data Availability

Proteomics data were deposited in the ProteomeXchange database (PXD029002) through partner MassIVE (MSV000088204; https://massive.ucsd.edu/ProteoSAFe/dataset.jsp?task=8bc139a2083f481fa7bcb0e62c8f1701). Other data generated or analyzed during this study are provided in the Supplementary Information and Source Data files. Source data are provided with this paper.

## Source data

Source Data

## Code availability

All code and input data are currently stored in Github (https://github.com/csbio/C.albicans-ml-pipeline) and Zenodo (10.5281/zenodo.5565593).
