# Supplementary material for: Leveraging machine learning essentiality predictions and chemogenomic interactions to identify antifungal targets
Source: Nat Commun. 2021 Nov 11;12:6497. doi: 10.1038/s41467-021-26850-3 (PMC8586148; doi:10.1038/s41467-021-26850-3)
Supplement: Supplementary file 1 — Supplementary Information [file 41467_2021_26850_MOESM1_ESM.pdf]

Supplementary Information

Leveraging machine learning essentiality predictions and chemogenomic interactions to identify antifungal targets

Supplementary Note 1

We note that there has been a previous effort to develop a machine learning (ML)-based model to predict gene essentiality in *C. albicans* (Segal *et al.*[1]). We provide an in-depth comparison of our approaches and results here. The differences between our approach and Segal *et al.*[1] are: (1) we used a different standard for training the ML approach: essentiality/non-essentiality was derived from the existing GRACE strains in our case, while the previous study used a standard derived from ortholog information; (2) we incorporated a broader set of gene features for predicting essentiality beyond the TnSeq features used by Segal *et al.*[1]. Specifically, we used the TnSeq features used in that study as well as gene expression, information from *S. cerevisiae* orthologs, CAI (codon adaptation index), and sequence conservation.

We performed some comparative analysis of the impact of these two differences. The relationship between our GRACE mutant library and the training standard set used by Segal *et al.*[1] is shown in Supplementary Fig. 2a. From the intersection between the two standards, we selected a training set of 806 genes where data for all 13 features and both standards were available (labeled GRACE_sub_train and Segal_sub_train). For the held-out test set, we picked 1,398 genes with all 13 features available from the remainder of GRACE standard (labeled GRACE_sub_test), and 493 genes with all 13 features available from the remainder of the Segal *et al.*[1] standard (labeled Segal_sub_test). Accordingly, we built random forest models[2] using all default parameters from scikit-learn[3], except for setting the number of estimators as 100 and the random state as 0 as is described by Segal *et al.*[1].

The goal of this analysis was to understand the effect of these differences in developing a generalizable model for predicting essential genes that could be confirmed using the GRACE

system. Based on these standards, we performed a series of evaluations, which are described in more detail below and in Supplementary Table 1:

Model A1: We reproduced the results with TnSeq input features and training standard from Segal *et al.*[1]. Our results of near-perfect prediction accuracy on the test set were consistent with their findings.

Model A2: We used the Segal *et al.*[1] dataset for training, but only tested on GRACE standard genes that are outside the original Segal *et al.*[1] training set (red part of the Venn diagram in Supplementary Fig. 2a). In this evaluation, the AUC dropped substantially, reflecting that the set of genes tested in the GRACE presents a more challenging prediction scenario than the training standard used for Segal *et al.*[1].

Model A3: We used Segal *et al.*[1] features but trained it on a gold standard defined by the GRACE set and tested it on Segal *et al.*[1] standard (green part of the Venn diagram in Supplementary Fig. 2a). We observed equally high performance as scenario #1, suggesting that the model performs well because the Segal *et al.*[1] gene standard encompasses easier to predict essential genes.

Model A4: We used Segal *et al.*[1] features but trained it on a gold standard defined by the GRACE set and tested it on the GRACE standard. This scenario yielded an AUC similar to scenario #2, substantially less than the performance on the original Segal *et al.*[1] test set.

We also repeated all of the above experiments with the full set of 13 features used in our study. For all scenarios (model B1, B2, B3, B4), the features beyond TnSeq (gene expression, information from *S. cerevisiae* orthologs, CAI, and sequence conservation) provided a boost in the performance, regardless of the gold standard used, suggesting that these features contribute unique information to prediction performance that is not captured by the TnSeq features. For example, with the full set of features, and a fully optimized random forest model (model B5) trained and tested on the whole original set of GRACE gold standard, we measured an AUC of 0.92 ± 0.01 and AP of 0.77 ± 0.04 relative to the TnSeq features-only model (A4), which achieved an AUC of 0.84 ± 0.02 and AP 0.40 ± 0.04. Overall, this suggests the additional features substantially improve prediction performance, especially when one uses a standard based on systematic construction of strains (i.e. the GRACE standard). This analysis also highlights the choice of gold standard for essential and non-essential genes dramatically affects both the nature of the models trained and their apparent performance during evaluation (compare Model A2 to A4 or B2 to B4). It is important to note that, as discussed in the main manuscript, our analysis suggests there may be an increased rate of false negatives in the GRACE library for essential genes with low expression. This bias introduced by the experimental system of course influences evaluations based on this standard, and is also learned by the machine learning models that rely on it for training.

Supplementary Table 1: Comparison of the performance on different features sets and standards used for training and testing random forest models.

Feature set	Model label	Train standard	Test standard	AUC	AP
**6 Segal *et al.* features**	A1	Segal_sub_train	Segal_sub_test	0.998 ± 0.001	0.991 ± 0.006
	A2	Segal_sub_train	GRACE_sub_test	0.84 ± 0.02	0.33 ± 0.03
	A3	GRACE_sub_train	Segal_sub_test	0.994 ± 0.002	0.972 ± 0.011
	A4	GRACE_sub_train	GRACE_sub_test	0.84 ± 0.02	0.40 ± 0.04
**13 integrated features**	B1	Segal_sub_train	Segal_sub_test	0.999 ± 0.0002	0.999 ± 0.001
	B2	Segal_sub_train	GRACE_sub_test	0.85 ± 0.02	0.39 ± 0.04
	B3	GRACE_sub_train	Segal_sub_test	0.999 ± 0.001	0.997 ± 0.002
	B4	GRACE_sub_train	GRACE_sub_test	0.88± 0.01	0.49 ± 0.04
	B5	$GRACE*_{optimal}$	$GRACE*_{optimal}$	0.92 ± 0.01	0.77 ± 0.04

***: The optimal model performance from the cross-validation analysis on the whole GRACE set**

Supplementary Table 2: Strains used in this study.

**Strain ID**	Description	Genotype	Source
**CaLC6106**	*C. albicans* CaSS1 (GRACE library parent)	*ura3::imm434/ura3::imm434 his3::hisG/his3::hisG leu2::tetRGAL4AD-URA/LEU2*	[4]
**GRACE strain, *SPC19***	*tetO-SPC19/spc19Δ*	As CaSS1, *SAT1::tetO-SPC19/SPC19::HIS3*	[4]
**GRACEv2 strain, *DAM1***	*tetO-DAM1/dam1Δ*	As CaSS1, *SAT1::tetO-DAM1/DAM1::HIS3*	This study
**GRACE strain, *DAD1***	*tetO-DAD1/dad1Δ*	As CaSS1, *SAT1::tetO-DAD1/DAD1::HIS3*	[4]
**GRACE strain, *DAD2***	*tetO-DAD2/dad2Δ*	As CaSS1, *SAT1::tetO-DAD2/DAD2::HIS3*	[4]
**GRACEv2 strain, *ASK1***	*tetO-ASK1/ask1Δ*	As CaSS1, *SAT1::tetO-ASK1/ASK1::HIS3*	This study
**GRACEv2 strain, *C1_01070C***	*tetO-C1_01070C/C1_01070CΔ*	As CaSS1, *SAT1::tetO-C1_01070C/C1_01070C::HIS3*	This study
**GRACE strain, *NSL1***	*tetO-NSL1/nsl1Δ*	As CaSS1, *SAT1::tetO-NSL1/NSL1::HIS3*	[4]
**GRACE strain, *NNF1***	*tetO-NNF1/nnf1Δ*	As CaSS1, *SAT1::tetO-NNF1/NNF1::HIS3*	[4]
**GRACEv2 strain, *MTW1***	*tetO-MTW1/mwt1Δ*	As CaSS1, *SAT1::tetO-MTW1/MTW1::HIS3*	This study
**GRACEv2 strain, *DUO1***	*tetO-DUO1/duo1Δ*	As CaSS1, *SAT1::tetO-DUO1/DUO1::HIS3*	This study
**GRACEv2 strain, *DAD3***	*tetO-DAD3/dad3Δ*	As CaSS1, *SAT1::tetO-DAD3/DAD3::HIS3*	This study
**GRACEv2 strain, *DAD4***	*tetO-DAD4/dad4Δ*	As CaSS1, *SAT1::tetO-DAD4/DAD4::HIS3*	This study
**GRACEv2 strain, *HSK3***	*tetO-HSK3/hsk3Δ*	As CaSS1, *SAT1::tetO-HSK3/HSK3::HIS3*	This study
**GRACEv2 strain, *SPC34***	*tetO-SPC34/spc34Δ*	As CaSS1, *SAT1::tetO-SPC34/SPC34::HIS3*	This study
**CaLC239**	*C. albicans*, SN95	*arg4 /arg4 his1 /his1 URA3/ura3 ::imm434 IRO1/iro1 ::imm434*	[5]
**CaLC7334**	*C1_01070C*-GFP/*C1_01070C*-GFP	As SN95, *C1_01070C*-GFP-NAT/*C1_01070C*-GFP-NAT	This study
**CaLC4449**	*ENO1/ENO1*-GFP	As SN95, *ENO1/ENO1*-GFP-NAT	[6]

**CaLC7411**	*C1_01070C*-GFP/*C1_01070C*-GFP *DAD1*-RFP/*DAD1*-RFP	As SN95, *C1_01070C*-GFP-NAT/*C1_01070C*-GFP-NAT *DAD1*-RFP-*ARG*/*DAD1*-RFP-*ARG*	This study
**CaLC7410**	*C1_01070C*-GFP/*C1_01070C*-GFP *MTW1*-RFP/*MTW1*-RFP	As SN95, *C1_01070C*-GFP-NAT/*C1_01070C*-GFP-NAT *MTW1*-RFP-*ARG*/*MTW1*-RFP-*ARG*	This study
**GRACEv2 strain, *C6_03200W***	*tetO-C6_03200W/C6_03200W*∆	As CaSS1, *SAT1::tetO-C6_03200W/C6_03200W::HIS3*	This study
**GRACE strain, *TIM12***	*tetO-TIM12/tim12Δ*	As CaSS1, *SAT1::tetO-TIM12/TIM12::HIS3*	[4]
**CaLC7321**	*C6_03200W*-GFP/*C6_03200W*-GFP	As SN95, *C6_03200W*-GFP-NAT/*C6_03200W*-GFP-NAT	This study
**CaLC7408**	*C6_03200W*-GFP/*C6_03200W*-GFP *GCF1*-RFP/*GCF1*-RFP	As SN95, *C6_03200W*-GFP-NAT/*C6_03200W*-GFP-NAT *GCF1*-RFP-*ARG*/*GCF1*-RFP-*ARG*	This study
**GRACEv2 strain, *GCF1***	*tetO-GCF1/gcf1Δ*	As CaSS1, *SAT1::tetO-GCF1/gcf1::HIS3*	This study
**GRACE strain, *RPG1***	*tetO-RPG1/rpg1Δ*	As CaSS1, *SAT1::tetO-RPG1/RPG1::HIS3*	[4]
**GRACE strain, *NIP1***	*tetO-NIP1/nip1Δ*	As CaSS1, *SAT1::tetO-NIP1/NIP1::HIS3*	[4]
**GRACE strain, *TIF35***	*tetO-TIF35/tif35Δ*	As CaSS1, *SAT1::tetO-TIF35/TIF35::HIS3*	[4]
**GRACE strain, *TIF34***	*tetO-TIF34/tif34Δ*	As CaSS1, *SAT1::tetO-TIF34/TIF34::HIS3*	[4]
**GRACE strain, *C5_02660C***	*tetO-C5_02660C/C5_02660CΔ*	As CaSS1, *SAT1::tetO-C5_02660C/C5_02660C::HIS3*	[4]
**GRACEv2 strain, *C2_04370W***	*tetO-C2_04370W/C2_04370WΔ*	As CaSS1, *SAT1::tetO-C2_04370W/C2_04370W::HIS3*	This study
**GRACEv2 strain, *HCR1***	*tetO-HCR1/hcr1Δ*	As CaSS1, *SAT1::tetO-HCR1/HCR1::HIS3*	This study
**GRACEv2 strain, *C3_07420W***	*tetO-C3_07420W/C3_07420WΔ*	As CaSS1, *SAT1::tetO-C3_07420W/C3_07420W::HIS3*	This study
**CauLC3438**	*Candida auris* (VPCI 673/P/12)	Clinical isolate	Gift from Anuradha Chowdhary

**CgLC3452**	*Candida glabrata* (F27)	Clinical isolate	Gift from Anuradha Chowdhary
**GRACE strain, *GLN4***	*tetO-GLN4/gln4Δ*	As CaSS1, *SAT1::tetO-GLN4/GLN4::HIS3*	[4]
**CaLC2749**	*C. albicans,* DSY1024 (Selection Parent)	*cdr1 hisG/cdr1 ::hisG cdr2 ::hisG/cdr2 ::hisG camdr1 ::hisG/camdr1 ::hisG flu1 ::hisG/flu1 ::hisG-URA3-hisG*	[7]
**CaLC7316**	R1 Gln4/Gln4^{M496L}	As DSY1024, Gln4/Gln4^{M496L}	This study
**CaLC7319**	R2 Gln4/Gln4^{W513S}	As DSY1024, Gln4/Gln4^{W513S}	This study
**CaLC7320**	R3 Gln4/Gln4^{W513C}	As DSY1024, Gln4/Gln4^{W513C}	This study
**CaLC867**	*C. albicans* CaCi2-GFP	As CaCi2, *ENO1/ENO1-GFP-NAT*	[8]
**HEK 293T-Luci**	Mammalian cell line HEK 293T-Luci	Firefly luciferase-expressing HEK 293T cells	[9]
**HET strain, *orf19.3108***	*orf19.3108/orf19.3108Δ*	As CaSS1, *orf19.3108/orf19.3108::HIS3*	[10]
**HET strain, *GLN4***	*GLN4/gln4Δ*	As CaSS1, *GLN4/GLN4::HIS3*	[10]
**HET strain, *orf19.6539***	*orf19.6539/orf19.6539Δ*	As CaSS1, *orf19.6539/orf19.6539::HIS3*	[10]
**All other strains used in this manuscript are members of the GRACE, GRACEv2, or HET deletion collections**			

Supplementary Table 3: Plasmids used in this study.

Plasmid ID	Description	Source
**pLC763**	Ca-FLP-NAT-*tetO*	[11]
**pLC1251**	pUC19+*HIS3*	This study
**pLC963**	pV1393-1 (CaCas9/sgRNA entry expression vector, contains Nat^{R} gene, targeting *NEUT5L*)	[12]
**pLC1208**	RFP-*ARG*	This study
**pLC389**	GFP-NAT	[13]
**pLC1100**	pFA-3HA-ARG	[14]

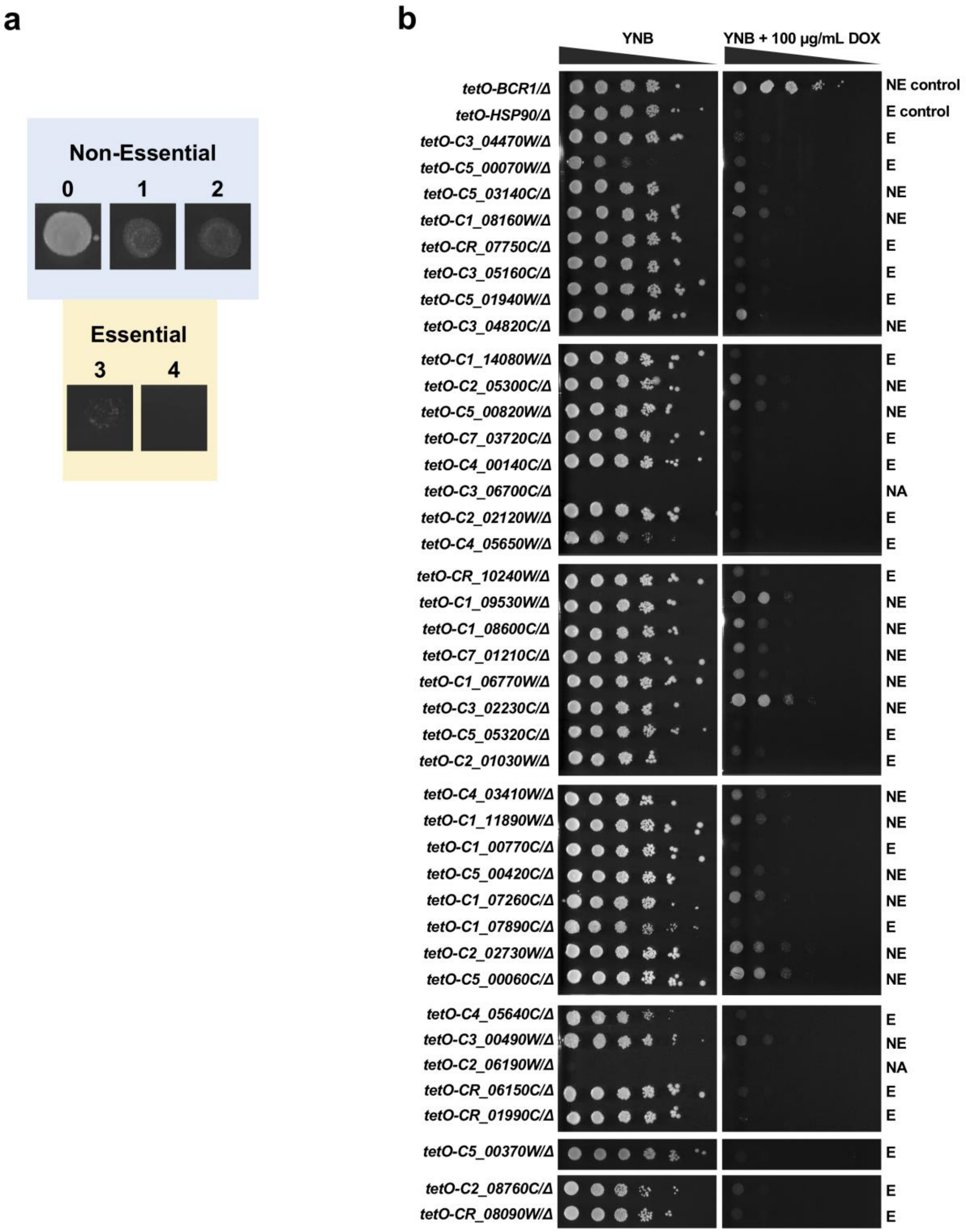

Supplementary Fig. 1: Scoring essentiality for the *C. albicans* GRACE and GRACEv2 collections. a) Schematic depicting scoring system that was used to score essentiality for strains

in the GRACE and GRACEv2 collections. Strains assigned a score of 3 or 4 by two independent researchers were classified as essential. **b)** Spotting validation for all GRACE and GRACEv2 strains that received discordant scores by the two independent researchers. The *tetO-BCR1/bcr1Δ* strain served as a non-essential (NE) control. The *tetO-HSP90/hsp90Δ* strain served as an essential control. Strains were grown overnight in the absence or presence of 0.05 μg/mL doxycycline (DOX) at which point they were spotted in a five-fold dilution (starting from an OD_{600} of 0.5) onto YNB agar alone or supplemented with 50 μg/mL DOX. Plates were photographed after growth for 48 hours at 30°C. Strains that did not grow in the absence of DOX could not be evaluated for essentiality (NA).

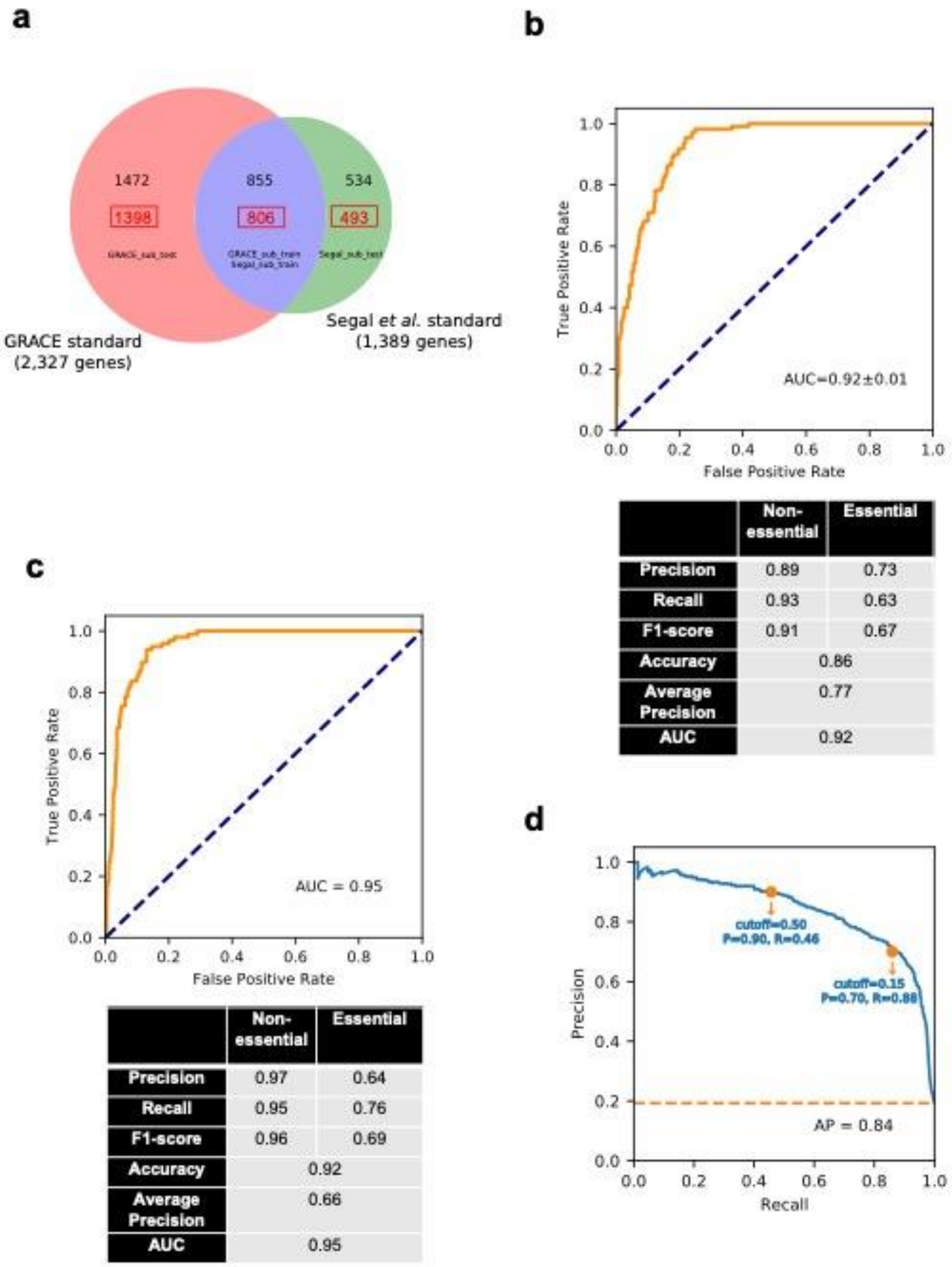

	Non-essential	Essential
Precision	0.89	0.73
Recall	0.93	0.63
F1-score	0.91	0.67
Accuracy	0.86	
Average Precision	0.77	
AUC	0.92	

	Non-essential	Essential
Precision	0.97	0.64
Recall	0.95	0.76
F1-score	0.96	0.69
Accuracy	0.92	
Average Precision	0.66	
AUC	0.95	

Supplementary Fig. 2: Assessment of machine learning model to predict essentiality. a) Venn

diagram highlighting the relationship between our GRACE standard and the Segal *et al.*[18] standard. Red boxes represent the subsets with all values available for the 13 features used as the training and testing sets in the Supplementary Analysis Section. **b)** Receiver Operating Characteristic (ROC) curve of our random forest model on the held-out 20% of the GRACE gene set. The model was trained and optimized on the other 80% of the GRACE gene set. The default stringent cutoff score for essential gene predictions results in an area under the ROC curve of 0.92. The error bars reflect the standard deviation across estimates derived from 10,000 different resamplings (with replacement) of the test set. **c)** Receiver Operating Characteristic (ROC) curve of the random forest model derived from the whole GRACE set and tested on the GRACEv2 experimental validation set. The default stringent cutoff score for essential gene predictions results in an area under the ROC curve of 0.95. d) Precision-recall curve that describes the recall at all possible prediction score cutoffs given the background of 1,278 known essential genes[21]. Thresholds corresponding to the stringent (RF score > 0.5) and the more relaxed (RF score > 0.15) points are starred.

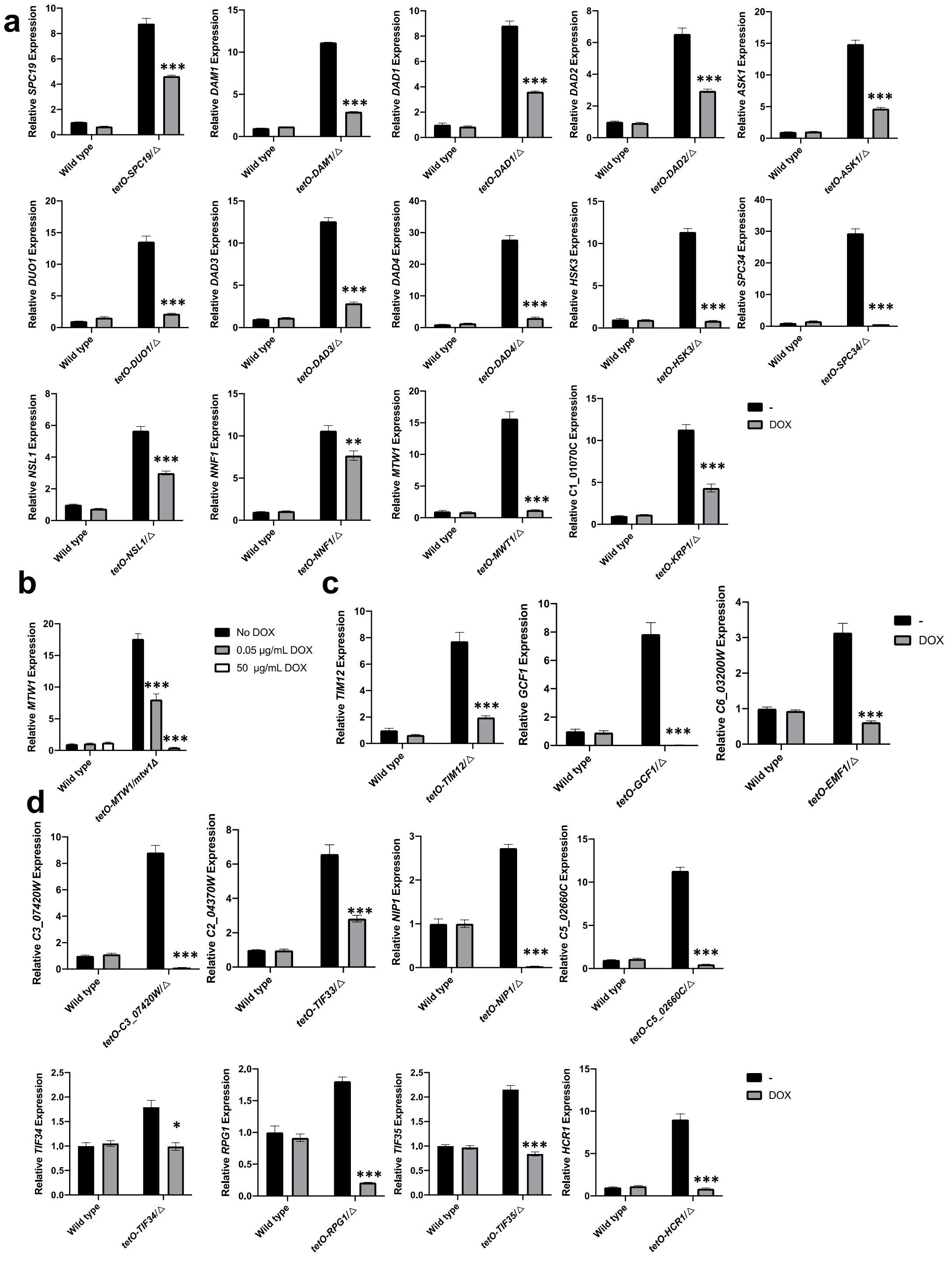

Supplementary Fig. 3: Quantification of transcript levels of prioritized GRACE strains.

Reverse transcriptase quantitative PCR (RT-qPCR) results for all GRACE and GRACEv2 strains characterized in **a-b)** Fig. 3 (DASH/Dam1 and MIND complexes) **c)** Fig. 4 (mitochondria-related genes) or **d)** Fig. 5 (translation initiation genes). YPD overnights of the wild-type strain and the GRACE strains were subcultured to an OD_{600} of 0.1 in YPD in the absence and presence of 0.05 µg/mL DOX for mitochondria-related genes, and the putative eIF3 subunit, or 0.1 µg/mL DOX for the Dam1/DASH and MIND complex GRACE strains (**a**). We also assessed transcriptional repression of the *tetO-MTW1/mtw1Δ* strain with 50 µg/mL DOX to ensure sufficient transcriptional repression in **b**. Relative level of expression for each gene was normalized to *GPD1* and *ACT1*. Bar graphs depict the mean of triplicate samples. Error bars, SEM. Significance assessed using two-way ANOVA, Tukey's multiple comparisons test. * indicate significant reduction in expression of GRACE or GRACEv2 strain in the presence of DOX relative to the absence of DOX. * P=0.0013, ** P= 0.0041, *** P<0.001. Source data are provided as a Source Data file.

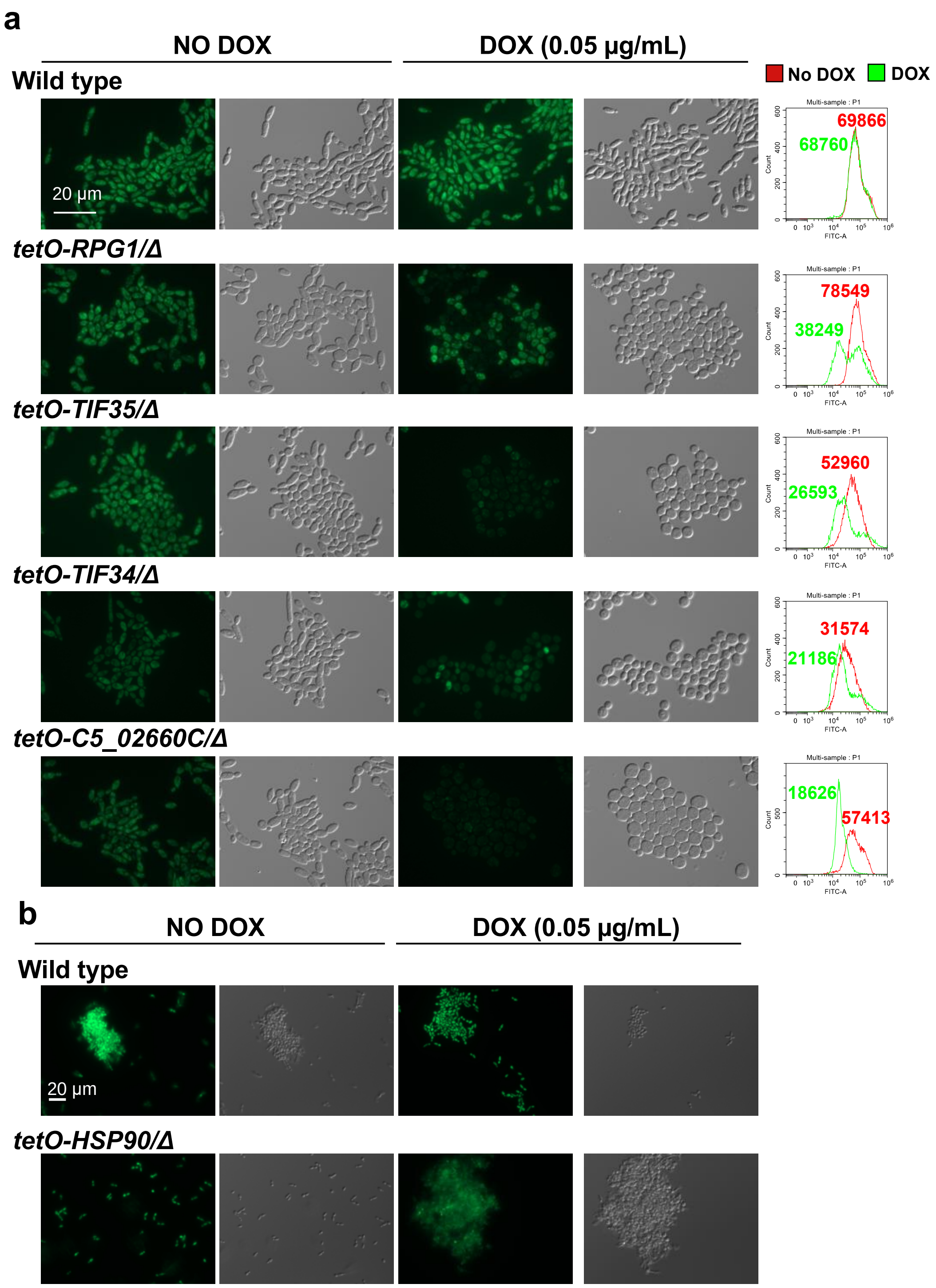
a
NO DOX
DOX (0.05 µg/mL)
No DOX
DOX
Wild type
20 µm
Multi-sample : P1
69866
68760
Count
FITC-A
tetO-RPG1/Δ
78549
38249
tetO-TIF35/Δ
52960
26593
tetO-TIF34/Δ
31574
21186
tetO-C5_02660C/Δ
18626
57413
b
NO DOX
DOX (0.05 µg/mL)
Wild type
20 µm
tetO-HSP90/Δ

Supplementary Fig. 4: Depletion of putative translation initiation genes impedes translation in *C. albicans.* **a)** A Click-iT protein synthesis assay kit was used to visualize protein translation. Strains were grown overnight in the absence or presence of 0.05 μg/mL DOX as indicated. Strains were subcultured to an OD_{600} of 0.1 in the same DOX conditions as the overnight and allowed to grow at 30°C for 4 hours. The l-homopropargylglycine (HPG) alkyne methionine analog was added, and then the cells were fixed. The azide fluorophore was added, and cells were imaged on the GFP channel to detect if translation had occurred. Cells were analyzed by flow cytometry. Histograms depict relative fluorescence intensity (FITC-A) of a minimum 20,000 events, values depict median fluorescence intensity (MFI). **b)** A Click-iT protein synthesis assay kit was used to visualize protein translation. Strains were grown as described in a before imaging by microscopy. All microscopy shown in this figure was performed in biological duplicate with similar results. Source data are provided as a Source Data file.

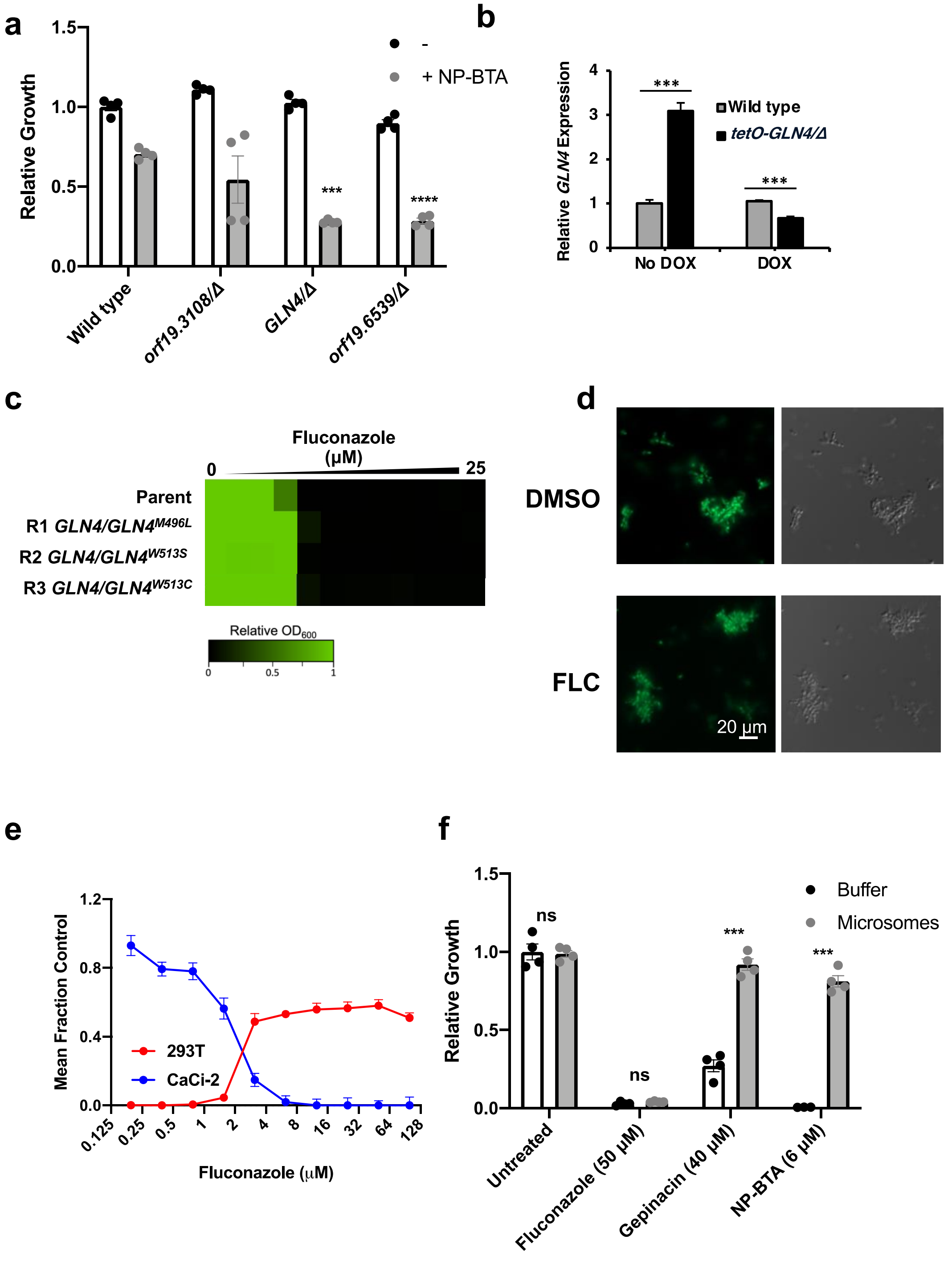

Supplementary Fig. 5: Characterization of NP-BTA as an inhibitor of *C. albicans* Gln4. a)

Confirmation of heterozygous mutant hypersensitivity to NP-BTA. Individual mutants identified by pooled screening were grown in the presence or absence of 1.2 μM NP-BTA for 24 hours in YPD at 30° C, and growth was measured every 15 minutes by OD_{600}. Relative growth was calculated by normalizing to the average AUC for the wild type no drug condition. Error bars represent SEM above and below the mean of technical quadruplicates. Significance assessed using two-way ANOVA, Bonferroni's multiple comparisons test, *** P<0.0003, **** P<0.0004 relative to wild type drug treated. Experiment was performed in biological duplicate with similar results obtained. **b)** YPD overnights of wild-type and *tetO-GLN4/gln4Δ* strains were subcultured to an OD_{600} of 0.1 in YPD in the absence and presence of 0.05 µg/mL DOX. Relative level of expression for each gene was normalized to *GPD1* and *ACT1*. Bar graphs depict the mean of triplicate samples. Error bars, SEM. Significance assessed using one-way ANOVA. *** indicates significant difference in expression of *tetO-GLN4/gln4Δ* relative to wild type, P<0.001. **c)** NP-BTA-resistant lineages are not resistant to fluconazole. Dose-response assays performed as described in Fig. 6. **d)** A Click-iT protein synthesis assay kit was used to visualize protein translation as described in Fig. 5. Cells were treated for 10 minutes with 25 μM of the antifungal fluconazole (FLC) or the DMSO solvent control, as indicated. Cells were analyzed by fluorescence microscopy. **e)** Fluconazole rescues mammalian cell growth in an *in vitro* co-culture model. Relative growth/survival of human kidney-derived cells (HEK293T-luciferase) and azole-tolerant *C. albicans* (CaCi-2-GFP, CaLC867) in co-culture. Concentration-dependent rescue of human cells by NP-BTA is plotted in relationship to inhibition of fungal burden. Red line indicates relative number of GFP-marked fungi/well. Blue line indicates relative number of luciferase-marked human cells present in the same well. Each point depicts the mean of triplicate wells. Error bars, SEM. Four-parameter curve fitting was performed in Prism v8.4. **f)** NP-BTA is not stable in mouse

microsomes. Compounds were incubated at 37°C with 5% CO_2 for 1 hour with microsomes (0.5 mg/ml protein) and an NADPH-regenerating system, then diluted 1:4 in YPD medium previously inoculated with *C. albicans*. Labels indicate compound concentrations present in cultures, assuming no metabolism. Fluconazole was included as a stable antifungal control and gepinacin was included as a non-stable control. Cultures were incubated at 30°C for 24 hours, then fungal growth was assessed by OD_{600}. Data are means -/+ SEM of remaining inhibitory activity for two independent experiments performed in technical quadruplicate. *** indicates significant difference in growth between buffer and microsome conditions, two-way ANOVA, Bonferroni's multiple comparisons test, $P<0.0001$. Source data are provided as a Source Data file.

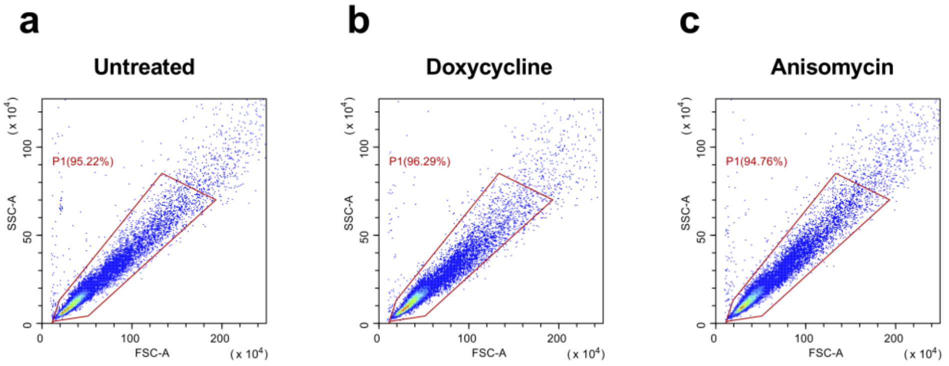

Supplementary Fig. 6: Summary of gating strategy from Click-it translation assay. Pseudo-coloured density plots displaying the side-scatter area and forward-scatter area for each even recorded in an untreated (**a**), 0.05 µg/mL doxycycline treated (**b**), and 100 µM anisomycin treated (**c**) sample of *C. albicans* cells when run on the Cytoflex flow cytometer and analyzed with the CytExpert Software. Gates displayed in red are the gates that were applied to all samples for data shown in panel Fig. 5d, Fig. 6f, and Supplementary Fig. 4.

Supplementary References:

1. Segal, E.S. *et al.* Gene essentiality analyzed by *in vivo* transposon mutagenesis and machine learning in a stable haploid isolate of *Candida albicans*. *mBio* **9**, e02048-18 (2018).
2. Breiman, L. Random Forests. *Machine Learning* **45**, 5-32 (2001).
3. Pedregosa, F. Scikit-learn: machine learning in Python. *J Mach Learn Res* **12**, 2825-2830 (2011).
4. Roemer, T. *et al.* Large-scale essential gene identification in *Candida albicans* and applications to antifungal drug discovery. *Mol Microbiol* **50**, 167-81 (2003).
5. Noble, S.M. & Johnson, A.D. Strains and strategies for large-scale gene deletion studies of the diploid human fungal pathogen *Candida albicans. Eukaryot Cell* **4**, 298-309 (2005).
6. O'Meara, T.R. *et al.* Global proteomic analyses define an environmentally contingent Hsp90 interactome and reveal chaperone-dependent regulation of stress granule proteins and the R2TP complex in a fungal pathogen. *PLoS Biol* **17**, e3000358 (2019).
7. Calabrese, D., Bille, J. & Sanglard, D. A novel multidrug efflux transporter gene of the major facilitator superfamily from *Candida albicans* (*FLU1*) conferring resistance to fluconazole. *Microbiology* **146 (Pt 11)**, 2743-54 (2000).
8. Whitesell, L. *et al.* Structural basis for species-selective targeting of Hsp90 in a pathogenic fungus. *Nat Commun* **10**, 402 (2019).

9. Stone, S.D., Lajkiewicz, N.J., Whitesell, L., Hilmy, A. & Porco, J.A., Jr. Biomimetic kinetic resolution: highly enantio- and diastereoselective transfer hydrogenation of aglain ketones to access flavagline natural products. *J Am Chem Soc* **137**, 525-30 (2015).

10. Xu, D. *et al.* Genome-wide fitness test and mechanism-of-action studies of inhibitory compounds in *Candida albicans*. *PLoS Pathog* **3**, e92 (2007).

11. Hossain, S., Veri, A.O. & Cowen, L.E. The proteasome governs fungal morphogenesis via functional connections with Hsp90 and cAMP-protein kinase A signaling. *mBio* **11**, e00290-20 (2020).

12. Veri, A.O. *et al.* Tuning Hsf1 levels drives distinct fungal morphogenetic programs with depletion impairing Hsp90 function and overexpression expanding the target space. *PLoS Genet* **14**, e1007270 (2018).

13. Gerami-Nejad, M., Berman, J. & Gale, C.A. Cassettes for PCR-mediated construction of green, yellow, and cyan fluorescent protein fusions in *Candida albicans*. *Yeast* **18**, 859-64 (2001).

14. Zhang, A. *et al.* The Tlo proteins are stoichiometric components of *Candida albicans* mediator anchored via the Med3 subunit. *Eukaryot Cell* **11**, 874-84 (2012).
