# Supplementary material for: Leveraging machine learning essentiality predictions and chemogenomic interactions to identify antifungal targets
Source: Nat Commun. 2021 Nov 11;12:6497. doi: 10.1038/s41467-021-26850-3 (PMC8586148; doi:10.1038/s41467-021-26850-3)
Supplement: Supplementary file 2 — Description of Additional Supplementary Files [file 41467_2021_26850_MOESM2_ESM.pdf]

Description of Additional Supplementary Files

File Name: Supplementary Data 1
Description: C. albicans essentiality predictions.

File Name: Supplementary Data 2
Description: Essentiality verdicts.

File Name: Supplementary Data 3
Description: GO Term Analysis.

File Name: Supplementary Data 4
Description: Fungal-specific essential genes.

File Name: Supplementary Data 5
Description: Putative cellular targets of pharmacologically active compounds.

File Name: Supplementary Data 6
Description: Mass spectrometry analysis; Krp1 interactome.

File Name: Supplementary Data 7
Description: Primers used in this study.
