# Supplementary material for: Leveraging machine learning essentiality predictions and chemogenomic interactions to identify antifungal targets
Source: Nat Commun. 2021 Nov 11;12:6497. doi: 10.1038/s41467-021-26850-3 (PMC8586148; doi:10.1038/s41467-021-26850-3)
Supplement: Supplementary file 10 — Reporting Summary [file 41467_2021_26850_MOESM10_ESM.pdf]

nature research

Corresponding author(s): Chad L. Myers, and Leah E. Cowen

Last updated by author(s): October 8, 2021

Reporting Summary

Nature Research wishes to improve the reproducibility of the work that we publish. This form provides structure for consistency and transparency in reporting. For further information on Nature Research policies, see our Editorial Policies and the Editorial Policy Checklist.

Statistics

For all statistical analyses, confirm that the following items are present in the figure legend, table legend, main text, or Methods section.

n/a	Confirmed	
☐	☒	The exact sample size (n) for each experimental group/condition, given as a discrete number and unit of measurement
☐	☒	A statement on whether measurements were taken from distinct samples or whether the same sample was measured repeatedly
☐	☒	The statistical test(s) used AND whether they are one- or two-sided<br>*Only common tests should be described solely by name; describe more complex techniques in the Methods section.*
☒	☐	A description of all covariates tested
☒	☐	A description of any assumptions or corrections, such as tests of normality and adjustment for multiple comparisons
☐	☒	A full description of the statistical parameters including central tendency (e.g. means) or other basic estimates (e.g. regression coefficient) AND variation (e.g. standard deviation) or associated estimates of uncertainty (e.g. confidence intervals)
☐	☒	For null hypothesis testing, the test statistic (e.g. F, t, r) with confidence intervals, effect sizes, degrees of freedom and P value noted<br>*Give P values as exact values whenever suitable.*
☒	☐	For Bayesian analysis, information on the choice of priors and Markov chain Monte Carlo settings
☒	☐	For hierarchical and complex designs, identification of the appropriate level for tests and full reporting of outcomes
☒	☐	Estimates of effect sizes (e.g. Cohen's d, Pearson's r), indicating how they were calculated

Our web collection on statistics for biologists contains articles on many of the points above.

Software and code

Policy information about availability of computer code

Data collection	Flow cytometry data was collected with the Beckman Coulter CytExpert Software (version 2.4).
Data analysis	All code and input data are currently stored in the following two repositories:<br>Github: https://github.com/csbio/C.albicans-ml-pipeline<br>Zenodo: https://doi.org/10.5281/zenodo.5565593<br>BioRad CFX Manager (version 3.1) was used to normalize RT-qPCR data and plot mean values with calculated SEM. GraphPad Prism (version 8.4.2) was used to plot bar graphs of RT-qPCR data and calculate all statistical significance using a standard two-tailed unpaired student t-test. Flow cytometry data was analyzed with the Beckman Coulter CytExpert Software (version 2.4). Java TreView (version 1.1.6r4) was used to generate all heat plots. Cytoscape (v3.8.2) was used to generate Krp1 interaction network. Predotar (version 1.04) was used to support predictions that Emf1 localized to the mitochondria.

For manuscripts utilizing custom algorithms or software that are central to the research but not yet described in published literature, software must be made available to editors and reviewers. We strongly encourage code deposition in a community repository (e.g. GitHub). See the Nature Research guidelines for submitting code & software for further information.

Data

Policy information about availability of data

All manuscripts must include a data availability statement. This statement should provide the following information, where applicable:

- Accession codes, unique identifiers, or web links for publicly available datasets
- A list of figures that have associated raw data
- A description of any restrictions on data availability

All data generated or analysed during this study are included in this published article (and its supplementary information files). Proteomics data was deposited in the ProteomeXchange database (PXD029002) through partner MassIVE (massive.ucds.edu; MSV000088204). (https://massive.ucsd.edu/ProteoSAFe/dataset.jsp?task=8bc139a2083f481fa7bcb0e62c8f1701).

Field-specific reporting

Please select the one below that is the best fit for your research. If you are not sure, read the appropriate sections before making your selection.

☒ Life sciences ☐ Behavioural & social sciences ☐ Ecological, evolutionary & environmental sciences

For a reference copy of the document with all sections, see nature.com/documents/nr-reporting-summary-flat.pdf

Life sciences study design

All studies must disclose on these points even when the disclosure is negative.

Sample size	Sample size was always n=3 or greater when statistical analysis was required. This was sufficient for data analysis given the limited variation observed between replicates. All experiments were performed in biological duplicate or greater with little deviation between replicates.
Data exclusions	In flow cytometry experiments, events were excluded from calculation of median fluorescence intensity (MFI) by gating on forward and side scatter parameters to eliminate debris and multi-cell clumps that would skew data. Gating removed less than 15% of all acquired events. No other data were excluded from analysis.
Replication	In cases of fungal growth assessment, assays were performed in technical duplicates which were averaged, in all other cases experiments were performed in technical triplicate as indicated in figure legends and methods. Each experiment was performed in at least biological duplicate with both replicates showing similar results.
Randomization	Randomization was used when scoring growth phenotypes of mutant strains on solid agar. The order which the strains were assessed were not decided based on phenotype. Randomization was not relevant to the other types of experimentation reported. All assays had a quantitative output that was generated through instrumentation (plate readers, RT-PCR machine, flow cytometer), rather than qualitative, and therefore, randomization was not required to eliminate user bias.
Blinding	Blinding was used when scoring growth phenotypes of mutant strains on solid agar. The authors did not know the strain ID when they were assessing growth. Blinding was not relevant to the other types of experimentation reported as there was no opportunity for bias to factor into quantitative results. All assays had a quantitative output that was generated through instrumentation (plate readers, RT-PCR machine, flow cytometer), rather than qualitative, and therefore, blinding was not required to eliminate user bias.

Reporting for specific materials, systems and methods

We require information from authors about some types of materials, experimental systems and methods used in many studies. Here, indicate whether each material, system or method listed is relevant to your study. If you are not sure if a list item applies to your research, read the appropriate section before selecting a response.

Materials & experimental systems

n/a	Involved in the study	
☒	☐	Antibodies
☐	☒	Eukaryotic cell lines
☒	☐	Palaeontology and archaeology
☐	☒	Animals and other organisms
☒	☐	Human research participants
☒	☐	Clinical data
☒	☐	Dual use research of concern

Methods

n/a	Involved in the study	
☒	☐	ChIP-seq
☐	☒	Flow cytometry
☒	☐	MRI-based neuroimaging

Eukaryotic cell lines

Policy information about cell lines

Cell line source(s)	Human HEK293T cells were obtained from American Type Culture collection (ATCC Cat# CRL-3216).
Authentication	Cell line was not authenticated as specific tissue of origin was non-critical to validity of results reported.
Mycoplasma contamination	All cell lines tested negative for mycoplasma contamination by PCR-based detection.
Commonly misidentified lines (See ICLAC register)	No commonly misidentified cell lines were used.

Animals and other organisms

Policy information about studies involving animals; ARRIVE guidelines recommended for reporting animal research

Laboratory animals	For mice experiments, 8- to 10-week-old female BALB/c mice (Charles River) were used. Rodent housing room temperature ranges of 69-74°F with 30-70% humidity. A 12 hour light /12 hour dark cycle was used.
Wild animals	No wild animals were used in this study.
Field-collected samples	No field-collected samples were used in this study.
Ethics oversight	Animal experiments were conducted with approval from UCSF Institutional Animal Care and Use Committee (protocol number AN189431-01).

Note that full information on the approval of the study protocol must also be provided in the manuscript.

Flow Cytometry

Plots

Confirm that:

☒ The axis labels state the marker and fluorochrome used (e.g. CD4-FITC).

☒ The axis scales are clearly visible. Include numbers along axes only for bottom left plot of group (a 'group' is an analysis of identical markers).

☒ All plots are contour plots with outliers or pseudocolor plots.

☒ A numerical value for number of cells or percentage (with statistics) is provided.

Methodology

Sample preparation	A 1:10 dilution of the C. albicans cell suspension was prepared in PBS in a flat bottom, transparent, 96-well plate (Beckman Coulter) to a final volume of 200 µL. Each sample was run using the CytExpert Software (version 2.4) until ~20,000 events had been recorded. Appropriate gating strategies were applied to all samples. Histograms show the FITC value for each event in a population following gating to exclude debris and doublets.
Instrument	Beckman Coulter CytoFlex Flow Cytometer - CytoFLEX S BC21021
Software	CytExpert Software
Cell population abundance	Populations were gated to remove debris and clumps for calculation of MFI. Populations reported represent >85% of total events.
Gating strategy	Gating was performed on the basis of forward and side scatter parameters to define a uniform single cell population and eliminate debris and multi-cell clumps that would skew data. Gated populations comprised >85% of all events acquired for all experiments.

☒ Tick this box to confirm that a figure exemplifying the gating strategy is provided in the Supplementary Information.
